# Supplementary material for: Identification and characterisation of vaginal bacteria-glycan interactions implicated in reproductive tract health and pregnancy outcomes
Source: Nat Commun. 2025 Jun 5;16:5207. doi: 10.1038/s41467-025-60404-1 (PMC12137855; doi:10.1038/s41467-025-60404-1)
Supplement: Supplementary file 1 — Supplementary Information [file 41467_2025_60404_MOESM1_ESM.pdf]

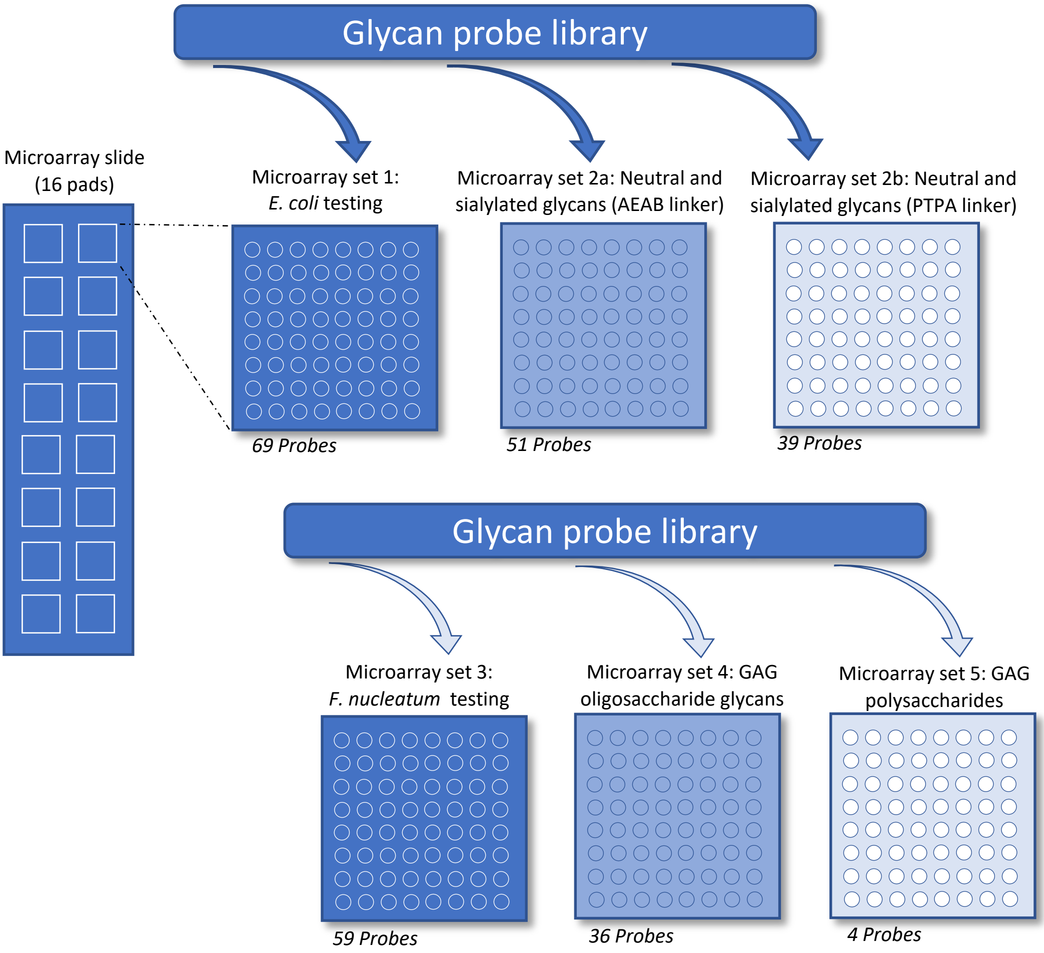

Supplementary Figure 1: Map of glycan microarray sets used in the study. Diagram representing the six glycan microarray sets used for analysis of whole bacteria binding to sequence-defined glycans. Microarray set 1 with a subset of 69 probes was used to investigate binding with four *E. coli* strains and for comparison of live and fixed *E. coli* C600 binding. Microarray set 3 was used for glycan binding comparison analysis of live and fixed *F. nucleatum* 23726 cultures. Microarray set 2a and 2b with a total of 90 neutral and sialylated glycans and Microarray set 4 with 36 GAG oligosaccharide probes were used to test the whole bacterial collection of 22 strains (Supplementary data 4) . Microarray set 5 with four GAG polysaccharide probes was used to test selected bacteria (Fig. 5B-C).

A

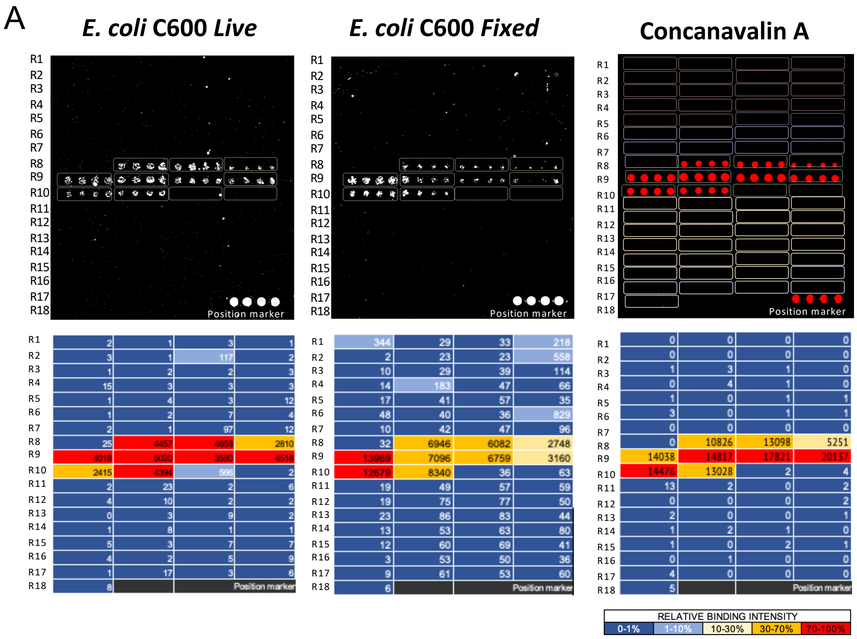

B

	E. coli C600 Assay_1			*E. coli* C600 Assay_2			*E. coli* C600 Assay_3			
Probe Name	MFI	SD	RANK	MFI	SD	RANK	MFI	SD	RANK	RANK 3
**Man5-GN2-AEAB**	4681	1348	71	13832	1139	100	10470	5608	100	90
**Man5-GN2-Gly**	6428	719	97	6887	923	50	9963	2444	95	81
Man6-GN2-AEAB	5266	1558	80	7139	3976	52	8754	2795	84	72
Man8-GN2-AEAB	5707	2618	86	12414	1159	90	3812	1780	36	71
Man9-GN2-AEAB	5702	1405	86	8750	4240	63	4309	2244	41	64
Man6-GN2-Gly	6619	1811	100	6130	197	44	4058	1384	39	61
Man7D1GN2-AEAB	5992	2520	91	6742	760	49	4527	736	43	61
Man7D3GN2-AEAB	2850	1325	43	3167	3778	23	1639	715	16	27
NA2-Gly	336	668	5	2738	1870	20	826	291	8	11

	E. coli 789 Assay_1			*E. coli* 789 Assay_2			*E. coli* 789 Assay_3			
Probe Name	MFI	SD	RANK	MFI	SD	RANK	MFI	SD	RANK	RANK 3
**Man6-GN2-AEAB**	7814	3442	85	7992	1573	100	6078	2983	71	86
**Man5-GN2-Gly**	7913	2849	87	7822	518	98	4937	1914	58	81
Man7D1GN2-AEAB	5494	562	60	6146	436	77	8530	1747	100	79
Man6-GN2-Gly	6572	3218	72	5631	258	70	6943	881	81	75
Man9-GN2-AEAB	9146	3722	100	5691	2823	71	3057	996	36	69
Man5-GN2-AEAB	6521	3099	71	7422	900	93	2541	372	30	65
Man8-GN2-AEAB	3384	2320	37	6602	698	83	2953	1071	35	51
Man7D3GN2-AEAB	1614	1038	18	4430	1105	55	5132	1270	60	44
NA2-Gly	786	848	9	2567	840	32	2546	427	30	24

	E. coli 901 Assay_1			*E. coli* 901 Assay_2			*E. coli* 901 Assay_3			
Probe Name	MFI	SD	RANK	MFI	SD	RANK	MFI	SD	RANK	RANK 3
**Man5-GN2-AEAB**	7881	1986	72	10084	988	100	14757	10894	100	91
**Man5-GN2-Gly**	10947	1683	100	8159	350	81	12068	4072	82	88
Man6-GN2-AEAB	3762	1761	34	7225	1099	72	8799	3348	60	55
Man7D1GN2-AEAB	3966	994	36	8380	1582	83	6600	2147	45	55
Man6-GN2-Gly	5814	523	53	7147	252	71	5643	617	38	54
Man8-GN2-AEAB	2445	957	22	5554	216	55	3740	773	25	34
Man9-GN2-AEAB	2349	795	21	5333	955	53	3429	2490	23	33
Man7D3GN2-AEAB	1885	506	17	5114	555	51	1534	850	10	26
NA2-Gly	20	24	0	1509	149	15	401	107	3	6

RELATIVE BINDING INTENSITY: 0-1% | 1-10% | 10-30% | 30-70% | 70-100%

Supplementary Figure 2. (A) Top: Microarray images of fluorescently labelled *E. coli* C600 live or fixed cells binding to glycans on Microarray set 1 (Supplementary Figure 1). Bottom: Quantified microarray data presented as heatmaps of relative binding intensities for each condition separately. Dark blue (0-1 %); Light blue (1-10 %); Yellow (10-30 %); Orange (30-70%); Red (70-100%). 100%, the maximum binding score observed for a given condition. Concanavalin A was used as a control for binding to oligo and high mannose N-glycans. (B) Table with mean fluorescence intensities (MFI), standard deviation of four replicates on the microarray (SD), RANK (or % of maximum intensity) and average rank of three independent binding assays (RANK3) of *E. coli* C600, 789 and 901 to the glycan Microarray set 1. The RANK for individual assays is calculated as 100 x Mean fluorescence intensity of probe/ Max mean fluorescence intensity in range of probes. Average rank of three assays (RANK3) is shown and coloured as in A. Source data are provided in Source Data file 3.

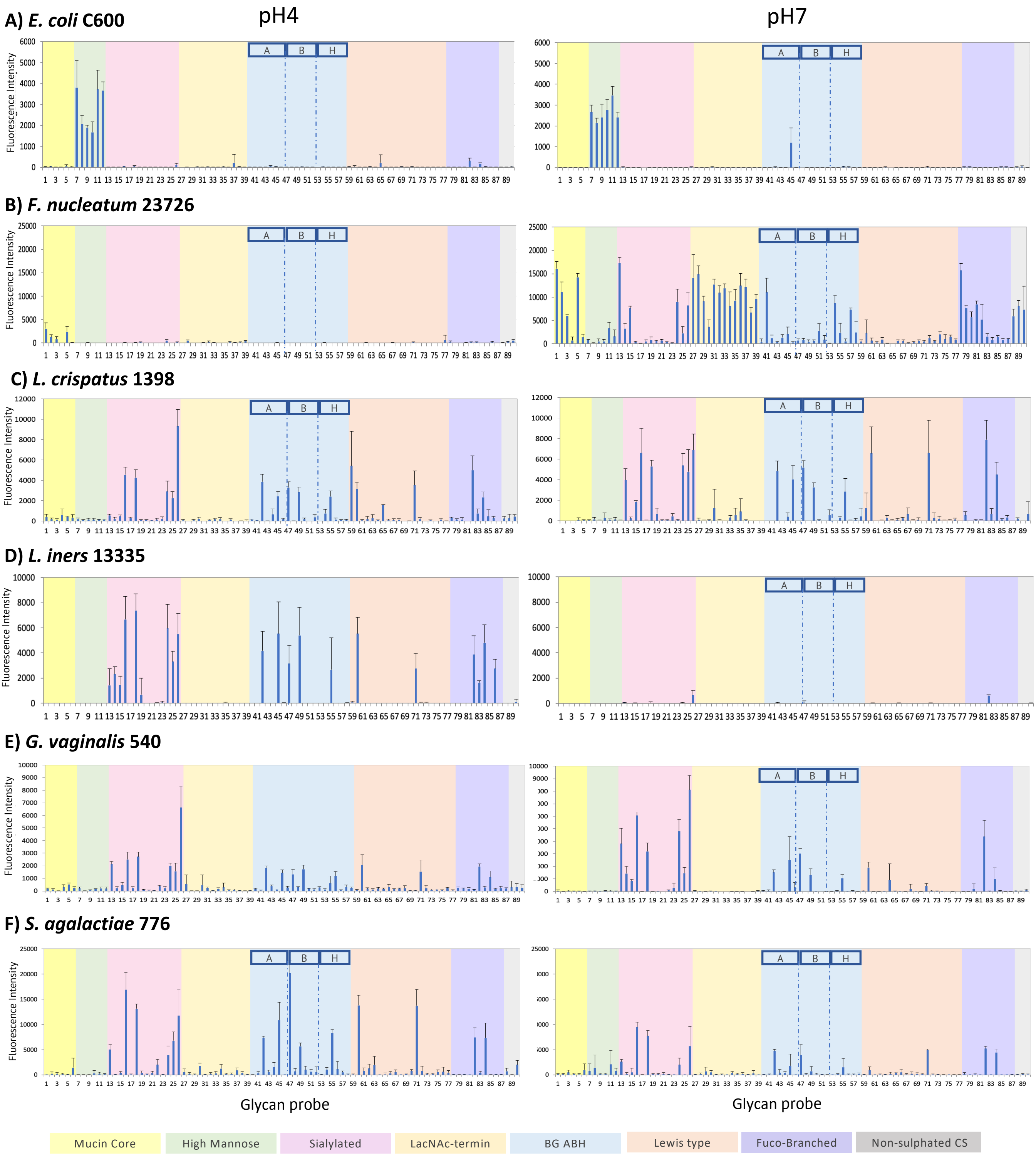

Supplementary Figure 3: Vaginal bacteria binding to neutral and sialylated glycans on sequence-defined covalent microarrays. Bar charts showing the binding fluorescence intensities to neutral and sialylated glycans at pH4 and pH7 of fixed cultures of A) *E. coli* C600 *and* B) *F. nucleatum* 23726 and live cultures of C) *L. crispatus* 1398*,* D) *L. iners* 13335*,* E) *G. vaginalis* 540 *and* F) *S. agalactiae* 776*.* Results are shown as mean fluorescence intensities of quadruplicate spots on high density (probes printed at 300 μM) Microarray set 2a and 2b (Supplementary Figure 1). Error bars represent SD of quadruplicate spots for each glycan probe (n=1). Glycan categories: Yellow: Mucin core related; Green: High Mannose related; Pink: Sialylated; Orange: LacNAc terminating; Blue: Blood group A, B, H glycans; Peach: Lewis blood group related; Purple: Fucosylated branched glycans from human milk; Grey: Non-sulphated chondroitin sulphate. The glycan sequences and structures corresponding to glycans 1-90 are included in Supplementary data 1. Glycan probe with index number #65 didn't pass quality control on Microarray set 2b. Source data are provided in Source Data file 4.

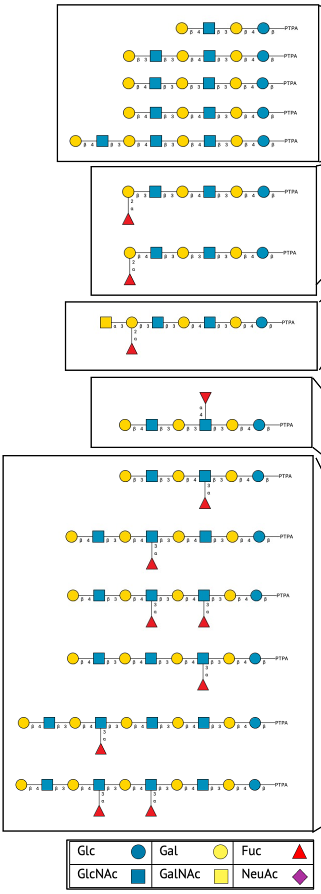

Index	Probe name	F. nucleatum 23726 pH4 Live. 100 uM	F. nucleatum 23726 pH4 Fixed 100 uM	F. nucleatum 23726 pH4 Fixed 300 uM	F. nucleatum 23726 pH7 Live 100 uM	F. nucleatum 23726 pH7 Fixed 100 uM	F. nucleatum 23726 pH7 Fixed 300 uM
30	Tetra T2-Lac-PTPA	355	472	36	11526	6966	3614
27	Hexa T1-T2-Lac-PTPA	-104	107	9	7184	4867	14128
31	Hexa T2-T1-Lac-PTPA	755	-104	77	9399	5867	12701
33	Hexa T2-T2-Lac-PTPA	905	352	136	9547	5017	11893
34	Octa T2-T2-T2-Lac-PTPA	797	606	47	12699	3944	8085
56	H Tetra T2-Lac-PTPA	927	463	27	407	90	492
54	H Hexa T1-T2-Lac-PTPA	-104	740	68	5631	2064	8685
57	H Hexa T2-T1-Lac-PTPA	356	360	42	7049	5296	7266
43	A Tetra T2-Lac-PTPA	-104	608	24	1062	291	371
41	A Hexa T1-T2-Lac-PTPA	237	379	57	7673	2739	11011
44	A Hexa T2-T1-Lac-PTPA	444	236	41	3233	1032	1343
50	B Tetra T2-Lac-PTPA	110	296	37	669	250	578
48	B Hexa T1-T2-Lac-PTPA	736	682	18	405	160	831
51	B Hexa T2-T1-Lac-PTPA	332	380	11	1251	2026	2748
61	Hexa T1(Leb)-T2-Lac-PTPA	134	507	27	142	63	679
62	A Hexa T1(Leb)-T2-Lac-PTPA	420	639	56	620	83	307
63	B Hexa T1(Leb)-T2-Lac-PTPA	449	414	53	564	369	941
65	Hexa T2(Ley)-T1-Lac-PTPA	971	181		245	113	
66	A Hexa T2(Ley)-T1-Lac-PTPA	347	549	44	310	95	600
67	B Hexa T2(Ley)-T1-Lac-PTPA	-104	388	70	263	591	572
69	Hexa T1(Lea)-T2-Lac-PTPA	283	523	7	2233	64	405
70	Hexa T1(Lea)-T2(Lex)-Lac-PTPA	519	337	9	208	492	592
32	Hexa T2-T1(Lea)-Lac-PTPA	462	273	157	9783	5760	10955
72	Hexa T2(Lex)-T1-Lac-PTPA	111	461	16	2729	100	1220
73	Hexa T2(Lex)-T1(Lea)-Lac-PTPA	258	129	22	528	97	554
74	Octa-T2(Lex)-T2-T2-Lac-PTPA	424	528	15	1303	375	2003
75	Octa-T2(Lex)-T2(Lex)-T2-Lac-PTPA	117	135	15	342	189	987
76	Octa-T2(Lex)-T2-T2(Lex)-Lac-PTPA	272	439	36	597	238	1496
77	Octa-T2(Lex)-T2(Lex)-T2(Lex)-Lac-PTPA	599	43	566	80	251	893
28	Hexa T1-T2(Lex)-Lac-PTPA	704	482	507	6706	6460	14894
35	Octa-T2-T2 (Lex)-T2-Lac-PTPA	687	756	32	8794	5206	9251
36	Octa-T2-T2(Lex)-T2(Lex)-Lac-PTPA	504	391	204	10239	5509	12478
37	Octa-T2-T2-T2(Lex)-Lac-PTPA	372	283	66	2914	3956	12144
38	Deca-T2-T2(Lex)-T2-T2-Lac-PTPA	121	18	151	5075	5164	6682
39	Deca-T2-T2(Lex)-T2(Lex)-T2-Lac-PTPA	640	-104	387	8645	4976	9587
19	3'Sia Hexa T2(Lex)-T1-Lac-PTPA	536	326	135	745	39	770
20	3'Sia Octa-T2(Lex)-T2-T2-Lac-PTPA	387	177	12	16	234	621
21	3'Sia Deca-T2-T2(Lex)-T2-T2-Lac-PTPA	85	49	12	56	21	712
22	3'Sia Deca-T2-T2(Lex)-T2(Lex)-T2-Lac-PTPA	186	82	33	96	141	453

RELATIVE BINDING INTENSITY				
0-1%	1-10%	10-30%	30-70%	70-100%

Supplementary Figure 4: **Comparison of glycan binding by fixed or live cultures *F. nucleatum* 23726.** Fluorescently labelled *F. nucleatum* 23726 live or fixed cultures were tested on Microarray set 3 (probes printed at 100 μM) and Microarray set 2b (300 μM) (Supplementary Figure 1). Heatmaps showing the mean fluorescence intensities of quadruplicates on microarrays at pH4 and pH7 are shown. Colour code: Dark blue (0-1 %); Light blue (1-10 %); Yellow (10-30 %); Orange (30-70%); Red (70-100%). Grey cells were flagged when they did not pass quality control in that microarray set (Probe index # 65 on microarray set 2b) or (-104) for artifact/background on slide. 100%, the maximum binding score observed for a given condition. Glycan structures corresponding to bound glycan probes are shown. Source data are provided in Source Data file 5.

A

Probe Name	*L. crispatus 1398* pH4 Acetate	pH4 HBS	pH7 Acetate	pH7 HBS	*L. iners 13335* pH4 Acetate	pH4 HBS	pH7 Acetate	pH7 HBS
CSA-DP6-AEAB	1148	1308	254	7	647	4311	16	102
CSA-DP10(3S)-AEAB	1361	2626	174	3	1353	5499	9	5
CSA-DP10(5S)-AEAB	3090	5781	10	13	5773	12336	14	1
CSA-DP14-AEAB	658	617	30	6	5455	6467	4	4
CSB-DP6(2S)-AEAB	548	835	11	5	312	989	9	6
CSB-DP6(3S)-AEAB	1476	2777	177	14	2114	5153	27	17
CSB-DP10(4S)-AEAB	2133	4210	6	5	5726	7677	1	1
CSB-DP10(5S)-AEAB	392	334	63	15	988	2463	11	7
CSB-DP14-AEAB	2592	3880	170	72	8178	12462	19	13
CSC-DP6-AEAB	2093	3644	57	6	3239	3439	10	9
CSC-DP10-AEAB	3336	6427	102	25	13410	10928	10	6
CSC-DP14-AEAB	601	962	16	13	1840	5340	35	11
Heparin-DP6-AEAB	737	1298	77	24	1002	1913	21	13
Heparin-DP10-AEAB	4309	5656	669	540	10325	6930	176	73
Heparin-DP14-AEAB	5497	8667	1106	686	17504	12002	361	72

Probe Name	*G. vaginalis 775* pH4 Acetate	pH4 HBS	pH7 Acetate	pH7 HBS	*S. agalactiae 776* pH4 Acetate	pH4 HBS	pH7 Acetate	pH7 HBS
CSA-DP6-AEAB	9462	11027	11	2	10301	9546	8	15
CSA-DP10(3S)-AEAB	1610	9773	5	10	9004	10718	46	2
CSA-DP10(5S)-AEAB	1396	12606	11	1	7475	21719	0	25
CSA-DP14-AEAB	748	13658	8	26	11225	17680	89	19
CSB-DP6(2S)-AEAB	3844	9655	12	4	6048	3148	6	3
CSB-DP6(3S)-AEAB	4193	6972	100	11	11735	16888	33	6
CSB-DP10(4S)-AEAB	495	11388	0	0	5889	16452	0	0
CSB-DP10(5S)-AEAB	578	8620	20	7	6689	5596	24	3
CSB-DP14-AEAB	10393	20189	19	18	22513	21093	148	75
CSC-DP6-AEAB	3878	11002	563	16	8795	18436	9	3
CSC-DP10-AEAB	1773	14140	16	2	11163	22608	127	2
CSC-DP14-AEAB	2250	13781	37	12	9418	24353	122	224
Heparin-DP6-AEAB	7266	13510	16	7	14891	17047	9	5
Heparin-DP10-AEAB	11325	12099	1849	61	23800	27118	118	10
Heparin-DP14-AEAB	2748	7515	566	22	19346	26627	170	10

Probe Name	*E. coli C600** pH4 Acetate	pH4 HBS	pH7 Acetate	pH7 HBS	*F. nucleatum 23726* pH4 Acetate	pH4 HBS	pH7 Acetate	pH7 HBS
CSA-DP6-AEAB	499	303	7	4	4510	4137	15	7
CSA-DP10(3S)-AEAB	330	108	7	3	4991	8950	565	1004
CSA-DP10(5S)-AEAB	584	215	0	0	9482	14603	3	227
CSA-DP14-AEAB	535	166	5	3	7912	8122	229	281
CSB-DP6(2S)-AEAB	327	92	4	2	3533	3945	176	33
CSB-DP6(3S)-AEAB	646	154	7	3	7888	9345	29	661
CSB-DP10(4S)-AEAB	452	127	1	0	8202	11765	16	574
CSB-DP10(5S)-AEAB	508	258	7	3	3764	4582	269	265
CSB-DP14-AEAB	544	179	8	4	10217	8719	262	68
CSC-DP6-AEAB	674	120	8	2	7117	6010	12	8
CSC-DP10-AEAB	674	212	2	2	8941	13495	16	215
CSC-DP14-AEAB	719	171	8	3	4674	7704	217	168
Heparin-DP6-AEAB	583	178	9	5	2267	3196	98	16
Heparin-DP10-AEAB	1216	434	51	6	8703	13778	403	976
Heparin-DP14-AEAB	1936	823	6	4	13956	16540	180	541

RELATIVE BINDING INTENSITY				
0-10%	10-25%	25-50%	50-70%	70-100%

B

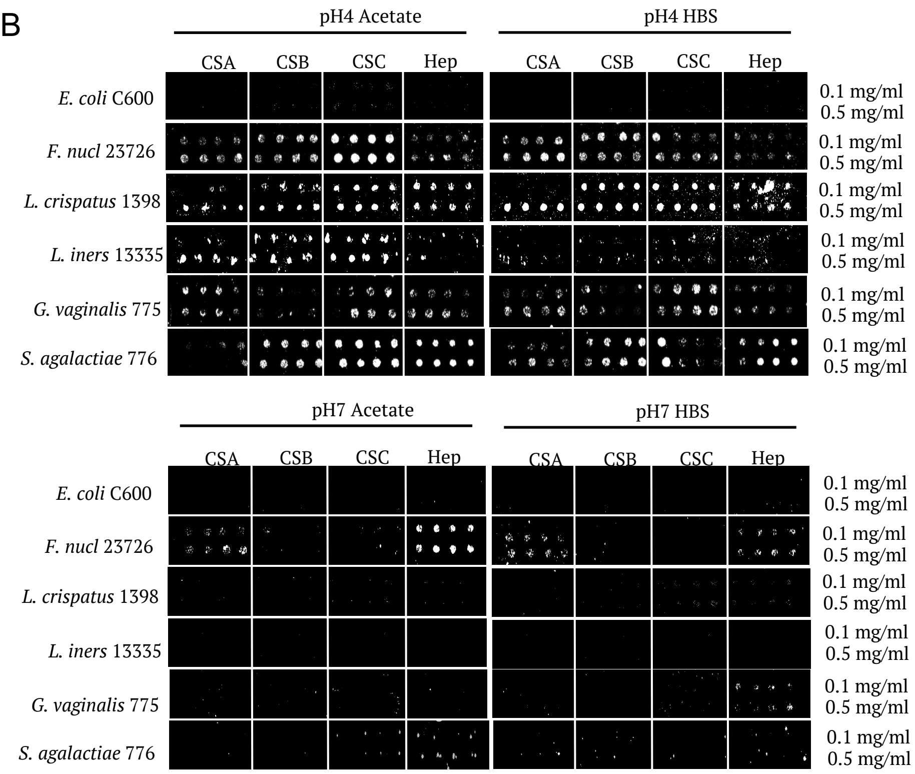

Glycans are printed in quadruplicate on arrays

Supplementary Figure 5. Glycosaminoglycan binding by bacteria of the vaginal microbiota in acetate and HBS buffers is pH dependent. (A) Heatmap showing the relative binding intensities to CS and Heparin oligosaccharides in acetate or HBS buffer and at pH4 and pH7 of live fluorescently labelled strains of *E. coli, F. nucleatum, L. crispatus, L. iners, G. vaginalis and S. agalactiae* on Microarray set 4 (Supplementary Figure 1) coloured as follows: Dark blue (0-10 %); Light blue (10-25 %); Yellow (25-50 %); Orange (50-70%); Red (70-100%). 100%, the maximum binding score observed for a given strain and condition. *Due to the low binding signals of *E. coli* C600 to GAGs in this experiment, a different colour palette has been applied for the heatmap: Dark blue (0-1 %); Light blue (1-10 %); Yellow (10-30 %); Orange (30-70%); Red (70-100%). Source data are provided in Source Data file 6. (B) Glycan microarray images of fluorescently labelled *E. coli* C600, *F. nucleatum* 23726, *L. crispatus* 1398, *L. iners* 13335, *G. vaginalis* 775 and *S. agalactiae* 776 binding to GAG polysaccharides in acetate or HBS buffer at pH4 or pH7. Assay is representative of two independent experiments.

A

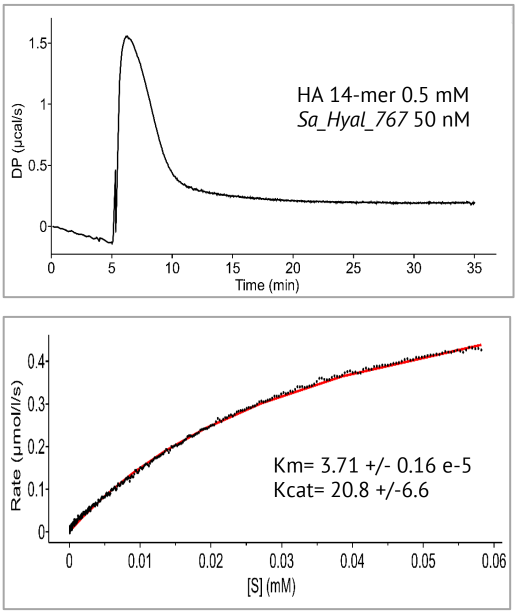

C

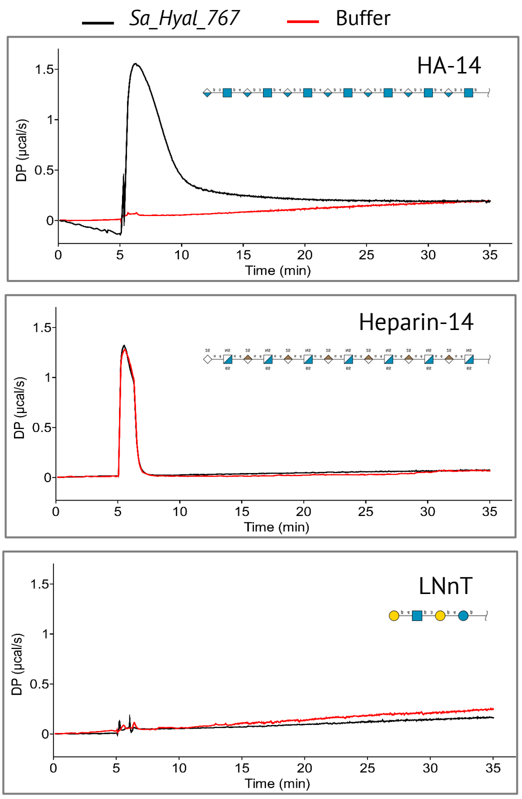

B

SAMPLE	Tᵉ (°C)	[Enzyme] (M)	[Substrate] (M)	ΔH (kcal/mol)	kcat (1/s)	KM (M)	Red. Chi-Sqr. (µmol/l/s)²
HA-14 pH 7.5	25.1	N/A	5.00E-04	N/A	N/A	N/A	N/A
Sa_Hyal_767 + HA14 pH7.5_001	25.1	5.00E-08	5.00E-04	16	12	3.59E-05	2.70E-05
Sa_Hyal_767 + HA14 pH7.5_002	25.1	5.00E-08	5.00E-04	18.9	17.5	3.84E-05	2.60E-05
Sa_Hyal_767 + HA14 pH7.5_003	25.1	5.00E-08	5.00E-04	14.1	29.4	3.55E-05	5.90E-05
Sa_Hyal_767 + HA14 pH7.5_ 004	25.1	5.00E-08	5.00E-04	18.3	24.5	3.86E-05	4.60E-05

Supplementary Figure 6: ITC shows that HA is a substrate of *S. agalactiae* 767 Hyaluronidase. (A) Representative thermal profile of a single injection experiment (top) and fitting to a Michaelis Menten curve (bottom) of calorimetry data generated following incubation of HA 14-mer with hyaluronidase from *S. agalactiae* 767 (*Sa_Hyal 767)* in the ITC cell. Black line (raw data), red line (fitted curve). (B) Table with *Km* and *Kcat* values inferred from four independent experiments. (C) Single injection isotherms generated following incubation of HA 14-mer, Heparin 14-mer and LNnT glycans with either buffer only (red line) or *Sa_Hyal 767 (*black line) in the ITC cell.

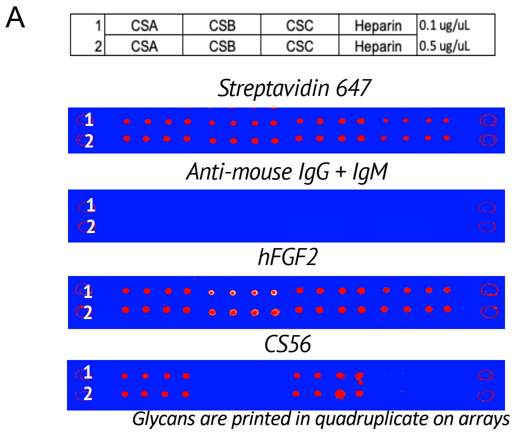

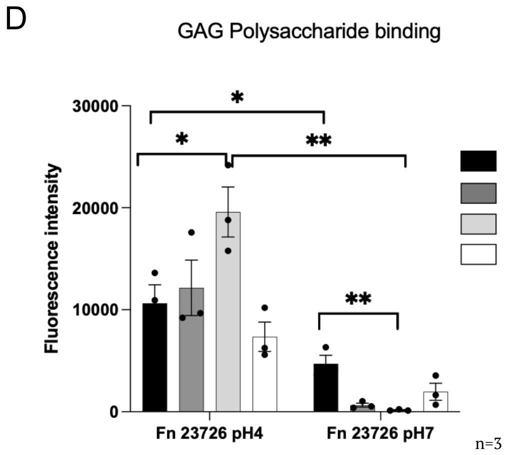

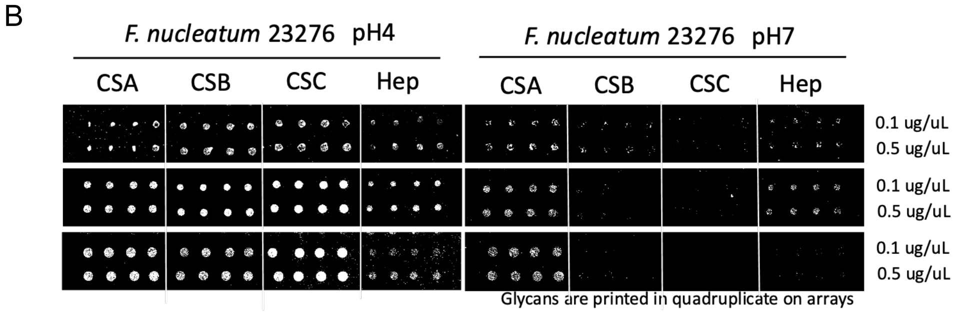

C

	F. nucleatum 23726 pH4 Assay 1				*F. nucleatum* 23726 pH4 Assay 2				*F. nucleatum* 23726 pH4 Assay 3				AVERAGE RANK of 3
Probe Name	Mean	SD	%CV	RANK	Mean	SD	%CV	RANK	Mean	SD	%CV	RANK	PH4
**CSC 0.5 ug/uL**	15779	1766	11	**90**	18799	3162	17	**100**	24178	3059	13	**100**	**97**
**CSC 0.1 ug/uL**	14155	3704	26	**80**	17007	4861	29	**90**	22829	3912	17	**94**	**88**
**CSB 0.5 ug/uL**	17585	2545	14	**100**	9219	1530	17	**49**	9660	1396	14	**40**	**63**
**CSB 0.1 ug/uL**	16424	1752	11	**93**	9610	863	9	**51**	8526	1006	12	**35**	**60**
**CSA 0.5 ug/uL**	10937	1279	12	**62**	7316	573	8	**39**	13614	2389	18	**56**	**52**
**CSA 0.1 ug/uL**	9121	2291	25	**52**	6964	1073	15	**37**	12297	2685	22	**51**	**47**
**Heparin 0.5 ug/uL**	10205	4243	42	**58**	6249	353	6	**33**	5594	401	7	**23**	**38**
**Heparin 0.1 ug/uL**	5824	2174	37	**33**	4616	703	15	**25**	3217	542	17	**13**	**24**

	F. nucleatum 23726 pH7 Assay 1				*F. nucleatum* 23726 pH7 Assay 2				*F. nucleatum* 23726 pH7 Assay 3				AVERAGE RANK of 3
Probe Name	Mean	SD	%CV	RANK	Mean	SD	%CV	RANK	Mean	SD	%CV	RANK	PH7
**CSA 0.5 ug/uL**	3603	1096	30	**100**	4197	428	10	**100**	6320	540	9	**100**	**100**
**CSA 0.1 ug/uL**	2505	646	26	**70**	4115	533	13	**98**	6321	590	9	**100**	**89**
**Heparin 0.5 ug/uL**	1636	480	29	**45**	3547	986	28	**85**	690	163	24	**11**	**47**
**Heparin 0.1 ug/uL**	1732	249	14	**48**	3113	771	25	**74**	709	163	23	**11**	**44**
**CSB 0.5 ug/uL**	1025	570	56	**28**	384	183	48	**9**	520	147	28	**8**	**15**
**CSB 0.1 ug/uL**	1017	102	10	**28**	311	230	74	**7**	378	234	62	**6**	**14**
**CSC 0.5 ug/uL**	136	98	72	**4**	280	159	57	**7**	131	14	11	**2**	**4**
**CSC 0.1 ug/uL**	123	36	29	**3**	249	71	28	**6**	114	4	3	**2**	**4**

Supplementary Figure 7: Chondroitin sulphate and heparin polysaccharides are bound by *F. nucleatum* 23726. (A) Top: Layout of the GAG polysaccharide Microarray set 5 containing biotinylated chondroitin sulphate A, B, C and Heparin probes printed in quadruplicate from 0.1 µg/µl and 0.5 µg/µl solutions. Bottom: Microarray images of polysaccharide binding by Streptavidin-Alexa647 or the indicated antibodies. (B) Microarray images from three independent experiments of fixed fluorescently labelled *F. nucleatum* 23726 binding at pH4 or pH7 to GAG polysaccharides. (C) Table with the analysis of the images 2 from B showing the background subtracted mean fluorescence intensities (Mean), SD, Coefficient of variance (%CV), and RANK (or % of MAX that is calculated as 100 x Mean for probe/Max Mean in range of probes). The average rank of three independent experiments is shown. (D) Bar charts show the average fluorescence intensities of *F. nucleatum* ATCC 23726 binding to GAG polysaccharides on glycan microarrays at pH4 and pH7. The average of the background-subtracted mean fluorescence intensities of bacterial binding from three independent experiments with quadruplicate spots printed using 0.5 µg/µl of polysaccharide on the microarray are shown. Error bars represent SD of n=3. * p<0.05, ** p<0.01 Unpaired t –test. Source data are provided in Source Data File 7.

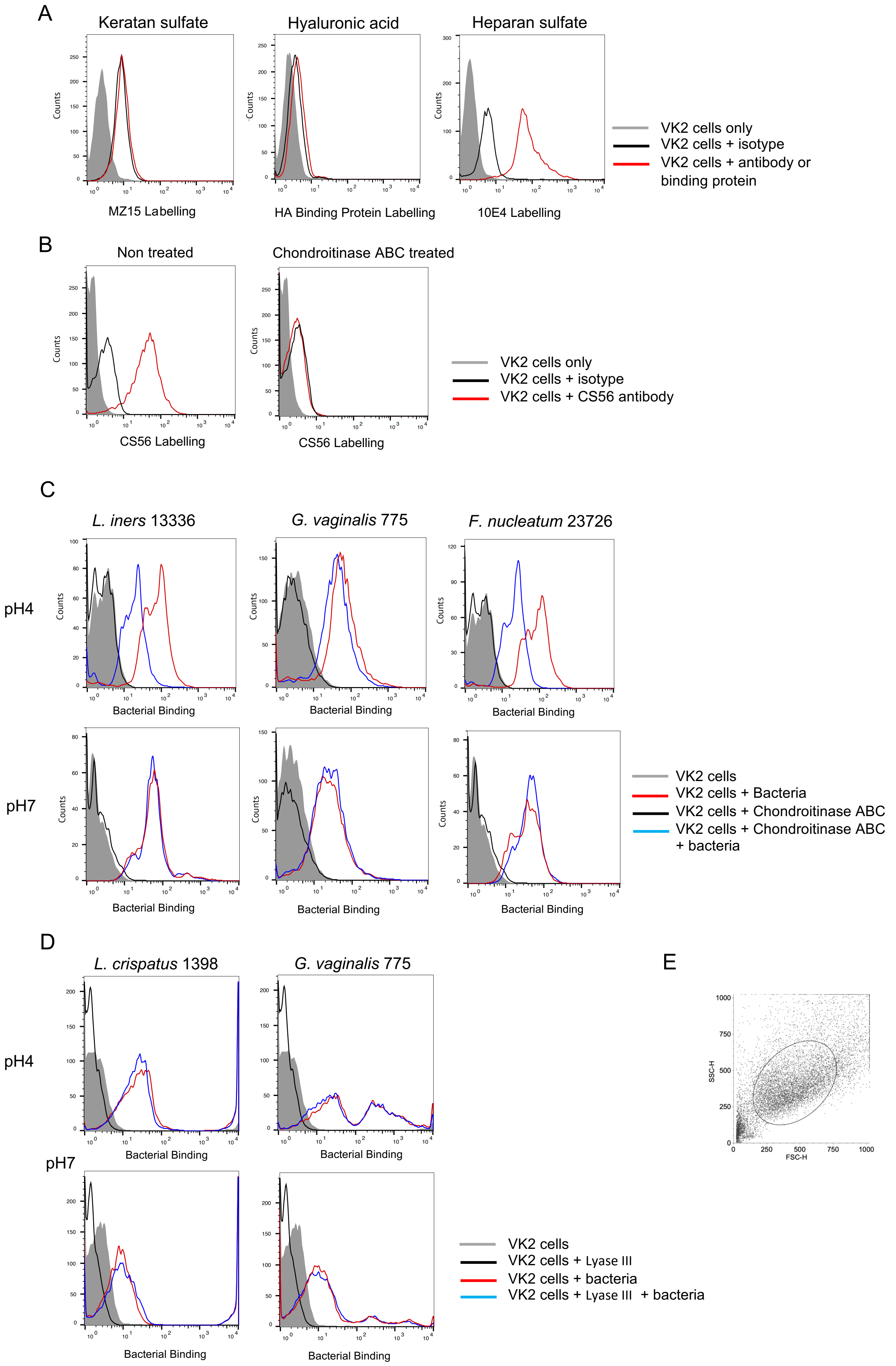
A
Keratan sulfate
Hyaluronic acid
Heparan sulfate
MZ15 Labelling
HA Binding Protein Labelling
10E4 Labelling
VK2 cells only
VK2 cells + isotype
VK2 cells + antibody or binding protein
B
Non treated
Chondroitinase ABC treated
CS56 Labelling
VK2 cells only
VK2 cells + isotype
VK2 cells + CS56 antibody
C
L. iners 13336
G. vaginalis 775
F. nucleatum 23726
pH4
pH7
Bacterial Binding
VK2 cells
VK2 cells + Bacteria
VK2 cells + Chondroitinase ABC
VK2 cells + Chondroitinase ABC + bacteria
D
L. crispatus 1398
G. vaginalis 775
pH4
pH7
Bacterial Binding
VK2 cells
VK2 cells + Lyase III
VK2 cells + bacteria
VK2 cells + Lyase III + bacteria
E
SSC-H
FSC-H
Counts

Supplementary Figure 8: Analysis of the expression of chondroitin sulphate, keratan sulphate, heparan sulphate and hyaluronic acid on vaginal epithelial VK2 cells. (A) VK2 cells were stained with the anti-keratan sulphate antibody MZ15, anti-heparan sulphate 10E4 antibody or HA binding protein and analysed by flow cytometry. (B) VK2 cells treated or not with chondroitinase were stained with the anti- CSA/CSC antibody CS56 and analysed by flow cytometry. (C) Flow cytometry-based analysis of live fluorescently labeled bacteria binding to VK2 cells treated or not with chondroitinase. (D) Flow cytometry-based analysis of fluorescently labeled bacteria binding to VK2 cells treated or not with Lyase III representative of three biological independent experiments. (E) Gating strategy applied to select the intact VK2 cells. Intact VK2 cells were gated based on their FFS and SSC.

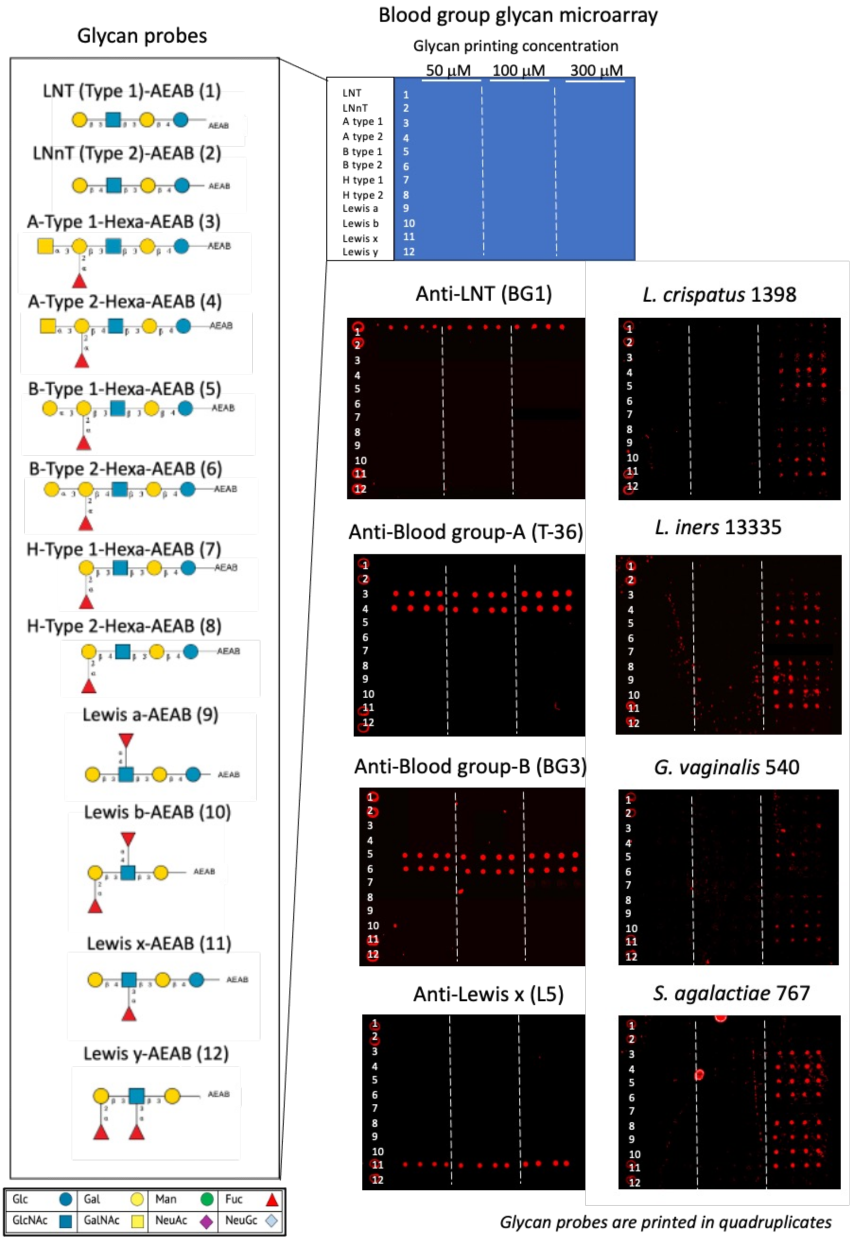

Supplementary Figure 9: Glycan density requirements for detecting binding of vaginal commensals and pathobionts to blood group glycans. Design of the blood group glycan microarray. The names of the 12 glycan probes and the corresponding glycan structures are shown. Three different glycan printing concentrations were used: 50, 100 and 300 µM. Microarray images of selected anti-carbohydrate antibodies and live fluorescently labelled bacteria binding are shown.

```
Met                 His tag
ATGGGCAGCAGCCATCATCATCATCATCACAGCAGCGGCCTGGTGCCGCGCGGCAGCCATATGGCTAGCATGAC
TGGTGGACAGCAAATGGGTCGCGGATCCATGGGTGACTTCAAGGAAAAGATCATCGACAAGAAAATTGATAAGA
AAAGCCAGTGGACCAACCTGTACGGCGCGAAGGACTGGAACACCTATATCGATCAGACCAAAAGCGTTAACAAG
AGCCCGATCATTCAACGTACCGAGCAGGGTCAAGTGAGCCTGAGCAGCGATAAGGGTTTTCGTGGCGCGGTGAC
CCAGAAAGTTAACATTGATCCGACCAAGAAATACGAAGTGAAGTTCGACATCGAGACCAGCAACAAAGTTGGTC
AAGCGTTTCTGCGTATTATGGAAAAGAAAGACAAAAACACCCGTCTGTGGCTGAGCGAGATGACCAGCGGCACC
ACCAACAAACACACCCTGACCAAGATCTATAACCCGAAACTGAACGTGAGCGAGGTTACCCTGGAACTGTACTA
TGAGAAGGGTACCGGCAGCGTTACCTTCGATAACATTAGCATGAAGGCGAAAGGTCCGAAAGACAGCGAACACC
CGCAGCCGGTGACCACCCAAATCGAGGAAAGCGTTAACACCGCGCTGAACAAGAACTACGTGTTTAACAAAGCG
GATTACCAGTATACCCTGACCAACCCGAGCCTGGGCAAGATCGTTGGTGGCATTCTGTATCCGAGCGCGACCGG
TAGCACCACCGTGAAAATCAGCGATAAGAGCGGCAAAATCATTAAGGAAGTGCCGCTGAGCGTTACCGCGAGCA
CCGAGGACAACTTCACCAAACTGCTGGACAAGTGGAACGATGTGACCATCGGTAACCACGTTTACGACACCAAC
GATAGCAACATGCAGAAACTGAACCAAAAGCTGGACGAGACCAACGCGAAAAACATCAAGGACATTAAACTGGA
TAGCAACCGTACCTTTCTGTGGGAAGATCTGAAGGGCCTGAACAACAGCGCGCAGCTGACCGCGACCTACCGTC
GTCTGGAGGACCTGGCGAAACAAATCACCAACCCGCACAGCACCATTTATAAGAACGAAAAAGCGATCCGTACC
GTTAAGGAGAGCCTGGCGTGGCTGCACCAGAACTTCTACAACGTGAACAAAGATATCGAAGGTAGCGCGAACTG
GTGGGACTTTGAGATCGGCGTTCCGCGTAGCATTACCGCGACCCTGGCGCTGATGAACAACTACTTCACCGACG
CGGAAATTAAGACCTATACCGATCCGATCGAGCACTTCGTGCCGGACGCGGGTTATTTTCGTAAGACCCTGGTT
AACCCGTTTAAAGCGCTGGGTGGCAACCTGGTGGATATGGGTCGTGTTAAGATCATTGAAGGCCTGCTGCGTAA
AGACAACACCATCATTAAGAAAACCAGCCACAGCCTGAAGAACCTGTTCACCACCGCGACCAAAGCGGAGGGTT
TTTACGCGGACGGCAGCTATATTGATCACACCAACGTGGCGTTCACCGGTGCGTACGGCAACGTTCTGATCGAC
GGTCTGACCCAGCTGCTGCCGATCATTCAAGAAACCGATTACAAGATTAGCAACCAGGAGCTGGACATGGTTTA
TAAATGGATTAACCAAAGCTTCCTGCCGCTGATCGTGAAGGGTGAACTGATGGATATGAGCCGTGGCCGTAGCA
TCAGCCGTGAAGCGGCGAGCAGCCATGCGGCGGCGGTTGAGGTGCTGCGTGGCTTTCTGCGTCTGGCGAACATG
AGCAACGAGGAACGTAACCTGGACCTGAAAAGCACCATTAAGACCATCATTACCAGCAACAAGTTCTACAACGT
TTTTAACAACCTGAAAAGCTATAGCGATATCGCGAACATGAACAAACTGCTGAACGACAGCACCGTGGCGACCA
AGCCGCTGAAAAGCAACCTGAGCACCTTCAACAGCATGGATCGTCTGGCGTACTATAACGCGGAGAAGGACTTC
GGTTTTGCGCTGAGCCTGCACAGCAAACGTACCCTGAACTACGAGGGTATGAACGATGAGAACACCCGTGGCTG
GTATACCGGTGATGGCATGTTCTACCTGTATAACAGCGACCAGAGCCACTACAGCAACCACTTTTGGCCGACCG
TTAACCCGTATAAGATGGCGGGTACCACCGAAAAAGACACCGGCCGTGAGGATACCATCAAGAAACTGATGAAC
CGTTACGACAAGACCAACAAAAACAGCAAGGTGATGACCGGTCAGGTTACCGGCACCAGCGATTTCGTGGGTAG
CGTTAAGCTGAACGACCACTTCGCGCTGGCGGCGATGGACTTTACCAACTGGGATCGTACCCTGACCGCGCAAA
AGGGTTGGGTGATCCTGAACGATAAAATTGTTTTTCTGGGCAGCAACATTAAAAACACCAACGGTGTGGGCAAC
GTTAGCACCACCATCGACCAGCGTAAAGACGATAGCAAGACCCCGTACACCACCTATGTGAACGGCAAGACCGT
TGATCTGAAACAAGCGAGCAGCCAGCAATTCACCGACACCAAGAGCGTGTTTCTGGAGAGCAAAGAACCGGGTC
GTAACATCGGCTACATTTTCTTTAAGAACAGCACCATCGACATTGAGCGTAAAGAACAGACCGGCACCTGGAAC
AGCATTAACCGTACCAGCAAGAACACCAGCATCGTTAGCAACCCGTTCATCACCATTAGCCAAAAACACGACAA
CAAGGGTGATAGCTACGGCTATATGATGGTGCCGAACATTGACCGTACCAGCTTTGATAAGCTGGCGAACAGCA
AAGAGGTGGAACTGCTGGAAAACAGCAGCAAGCAGCAAGTTATCTACGATAAAAACAGCCAGACCTGGGCGGTG
ATTAAACACGACAACCAGGAGAGCCTGATCAACAACCAATTCAAAATGAACAAGGCGGGTCTGTACCTGGTGCA
GAAGGTTGGCAACGATTATCAAAACGTGTACTATCAGCCGCAAACCATGACCAAAACCGACCAACTGGCGATCT
AA
```

Supplementary Figure 10: Nucleotide sequence used for the expression of *SaHyal_767.* The sequence specified encoding the *SaHyal_767* hyaluronate lyase from *Streptococcus agalactiae* strain 767 was codon optimized and cloned in the plasmid pET28a (+) for expression in *E. coli*. The recombinant full-length *SaHyal_767* enzyme includes the appended carbohydrate binding module (CBM70), and the catalytic module in which Tyr578 was mutated to Phe (highlighted in pink). The Y578F mutant was designed to reduce catalytic activity to perform glycan binding assays with the full-length enzyme (Supplementary Figure 6).
