# Supplementary material for: Identification and characterisation of vaginal bacteria-glycan interactions implicated in reproductive tract health and pregnancy outcomes
Source: Nat Commun. 2025 Jun 5;16:5207. doi: 10.1038/s41467-025-60404-1 (PMC12137855; doi:10.1038/s41467-025-60404-1)
Supplement: Supplementary file 2 — Description Of Additional Supplementary Data [file 41467_2025_60404_MOESM2_ESM.pdf]

Description of Additional supplementary files:

Supplementary Data 1:

List of glycan probes

Supplementary Data 2:

Anti-carbohydrate antibodies, plant lectins and glycan binding proteins

Supplementary Data 3:

MIRAGE document

Supplementary Data 4:

Bacterial strains
