# Supplementary material for: Identification and characterisation of vaginal bacteria-glycan interactions implicated in reproductive tract health and pregnancy outcomes
Source: Nat Commun. 2025 Jun 5;16:5207. doi: 10.1038/s41467-025-60404-1 (PMC12137855; doi:10.1038/s41467-025-60404-1)
Supplement: Supplementary file 7 — Reporting summary [file 41467_2025_60404_MOESM7_ESM.pdf]

Corresponding author(s): Virginia Tajadura-Ortega
David A MacIntyre
Yan Liu

Last updated by author(s): May 9, 2025

Reporting Summary

Nature Portfolio wishes to improve the reproducibility of the work that we publish. This form provides structure for consistency and transparency in reporting. For further information on Nature Portfolio policies, see our Editorial Policies and the Editorial Policy Checklist.

Statistics

For all statistical analyses, confirm that the following items are present in the figure legend, table legend, main text, or Methods section.

n/a	Confirmed	
☐	☒	The exact sample size (*n*) for each experimental group/condition, given as a discrete number and unit of measurement
☐	☒	A statement on whether measurements were taken from distinct samples or whether the same sample was measured repeatedly
☐	☒	The statistical test(s) used AND whether they are one- or two-sided<br>*Only common tests should be described solely by name; describe more complex techniques in the Methods section.*
☒	☐	A description of all covariates tested
☒	☐	A description of any assumptions or corrections, such as tests of normality and adjustment for multiple comparisons
☐	☒	A full description of the statistical parameters including central tendency (e.g. means) or other basic estimates (e.g. regression coefficient) AND variation (e.g. standard deviation) or associated estimates of uncertainty (e.g. confidence intervals)
☐	☒	For null hypothesis testing, the test statistic (e.g. F, t, r) with confidence intervals, effect sizes, degrees of freedom and P value noted<br>*Give P values as exact values whenever suitable.*
☒	☐	For Bayesian analysis, information on the choice of priors and Markov chain Monte Carlo settings
☒	☐	For hierarchical and complex designs, identification of the appropriate level for tests and full reporting of outcomes
☒	☐	Estimates of effect sizes (e.g. Cohen's *d*, Pearson's *r*), indicating how they were calculated

Our web collection on statistics for biologists contains articles on many of the points above.

Software and code

Policy information about availability of computer code

Data collection	GenePix 4300A was used for glycan miroarray data acquisition, Waters Q-TOF-type mass spectrometer SYNAPT-G2 and Axima MALDI Resonance mass spectrometer software for mass spectrometry data acquisition; CellQuest software was used for flow citometry data acquisition and MicroCal PEAQ-ITC calorimeter for ITC data acquisition. A Tecnai 12 Spirit transmission electron microscope (FEI) was used for acquisition of eletro-microscopy images.
Data analysis	GenePix® Pro 7 was used for glycan miroarray data visualization and quantitation and CarbarrayART v1 for data storage and analysis (Akune, Y. et al. CarbArrayART: a new software tool for carbohydrate microarray data storage, processing, presentation, and reporting. Glycobiology 32, 552-555 (2022). Available from https://glycosciences.med.ic.ac.uk/carbarrayart.html. MicroCal PEAQ-ITC Analysis Software (Malvern) was used for ITC data analysis. FlowJo 10.10.0 software was used for flow cytometry data analysis. Prism software (Graphpad Software, version 10.1.1) was used to perform statistical tests and to generate graphs.

For manuscripts utilizing custom algorithms or software that are central to the research but not yet described in published literature, software must be made available to editors and reviewers. We strongly encourage code deposition in a community repository (e.g. GitHub). See the Nature Portfolio guidelines for submitting code & software for further information.

Data

Policy information about availability of data

All manuscripts must include a data availability statement. This statement should provide the following information, where applicable:

- Accession codes, unique identifiers, or web links for publicly available datasets
- A description of any restrictions on data availability
- For clinical datasets or third party data, please ensure that the statement adheres to our policy

Source data and glycan microarray data are provided with this paper as Source Data Files. Original glycan microarray image files and gpr files are available upon request. Glycan microarray processed data is provided in Source Data Files 1-6. Glycan microarray metadata is summarized in Supplementary File 3 with MIRAGE information. There are no restrictions on the data availability.

Research involving human participants, their data, or biological material

Policy information about studies with human participants or human data. See also policy information about sex, gender (identity/presentation), and sexual orientation and race, ethnicity and racism.

Reporting on sex and gender

Use the terms sex (biological attribute) and gender (shaped by social and cultural circumstances) carefully in order to avoid confusing both terms. Indicate if findings apply to only one sex or gender; describe whether sex and gender were considered in study design; whether sex and/or gender was determined based on self-reporting or assigned and methods used.
Provide in the source data disaggregated sex and gender data, where this information has been collected, and if consent has been obtained for sharing of individual-level data; provide overall numbers in this Reporting Summary. Please state if this information has not been collected.
Report sex- and gender-based analyses where performed, justify reasons for lack of sex- and gender-based analysis.

Reporting on race, ethnicity, or other socially relevant groupings

Please specify the socially constructed or socially relevant categorization variable(s) used in your manuscript and explain why they were used. Please note that such variables should not be used as proxies for other socially constructed/relevant variables (for example, race or ethnicity should not be used as a proxy for socioeconomic status).
Provide clear definitions of the relevant terms used, how they were provided (by the participants/respondents, the researchers, or third parties), and the method(s) used to classify people into the different categories (e.g. self-report, census or administrative data, social media data, etc.)
Please provide details about how you controlled for confounding variables in your analyses.

Population characteristics

Describe the covariate-relevant population characteristics of the human research participants (e.g. age, genotypic information, past and current diagnosis and treatment categories). If you filled out the behavioural & social sciences study design questions and have nothing to add here, write "See above."

Recruitment

Describe how participants were recruited. Outline any potential self-selection bias or other biases that may be present and how these are likely to impact results.

Ethics oversight

Identify the organization(s) that approved the study protocol.

Note that full information on the approval of the study protocol must also be provided in the manuscript.

Field-specific reporting

Please select the one below that is the best fit for your research. If you are not sure, read the appropriate sections before making your selection.

☒ Life sciences ☐ Behavioural & social sciences ☐ Ecological, evolutionary & environmental sciences

For a reference copy of the document with all sections, see nature.com/documents/nr-reporting-summary-flat.pdf

Life sciences study design

All studies must disclose on these points even when the disclosure is negative.

Sample size

No sample size calculation was performed. For biological assays on human vaginal epithelial cells, n=3 was used and that was enough to achieve statistical significance in the comparison of the means.
For other validation assays, ie: ITC assays, several independent injections were carried out as described before (Hansen, L., Transtrum, M., Quinn, C. & Demarse, N. Enzyme-catalyzed and binding reaction kinetics determined by titration calorimetry. Biochimica et biophysica acta 1860, 957-966 (2016).
For microscopy assays, several fields of view were acquired that showed similar structures.
In glycan microarray binding analyses each assay was performed with four technical replicates, results in graphs are presented as mean +/- SD of quadruplicates (Supplementary tables 2-5). Most experiments were repeated with independent biological samples as described in the manuscript with similar results.

Data exclusions

Data corresponding to probe 65 that didn't pass QC in Microarray Set 2b was excluded for analysis on Figure 3 and Supplementary Figure 4 as

Data exclusions	stated in the figure legend and in the MIRAGE document (Supplementary File 3). For artifact on slides, data was flagged in GenPix and results shown as grey cells (-104) on the heatmaps. This was included in the figure legends in Figure 4 and Supplementary Figure 4.
Replication	Most experiments were repeated with independent biological samples as described in the manuscript with similar results. Additionally orthogonal biophysical, biochemical and biological assays were performed to validate the findings obtained on glycan microarrays.
Randomization	N/A
Blinding	Blinding was not used as the nature of the experiments didn't require it. For most bacterial binding on glycan microarray assays there are no controls groups. Acquisition of data was done simultaneously for all samples on glycan microarray assays.

Reporting for specific materials, systems and methods

We require information from authors about some types of materials, experimental systems and methods used in many studies. Here, indicate whether each material, system or method listed is relevant to your study. If you are not sure if a list item applies to your research, read the appropriate section before selecting a response.

Materials & experimental systems

n/a	Involved in the study
☐	☒ Antibodies
☐	☒ Eukaryotic cell lines
☒	☐ Palaeontology and archaeology
☐	☒ Animals and other organisms
☒	☐ Clinical data
☒	☐ Dual use research of concern
☒	☐ Plants

Methods

n/a	Involved in the study
☒	☐ ChIP-seq
☐	☒ Flow cytometry
☒	☐ MRI-based neuroimaging

Antibodies

Antibodies used

A comprehensive list of the primary antibodies used in this work is provided in Supplementary file 2, with clone number when applicable, dilution and source.

Other detection antibodies used:
Goat anti-mouse IgM, biotinylated (Vector Lab - BA2020 - polyclonal- LOT#Y1102)
Rabbit anti-rat IgM, biotinylated (Rockland Immunochemicals - 612-4607 – polyclonal – LOT#14623)
Goat anti-rabbit IgG, biotinylated (Sigma-Aldrich Merck - B7389 – polyclonal - LOT#SLBM7718V)
Goat anti-human IgG, biotinylated (Vector Lab - BA3000- polyclonal - LOT#ZA0316)
Alexa Fluor 488-conjugated anti-mIgG (A-11001, ThermoFisher)
Alexa Fluor 647-conjugated goat anti-mIgM antibodies (A-21238, ThermoFisher)

Detection antibodies were used 1:200 on glycan microarrays.

Validation

The specificity of all the primary and secondary antibodies used for the recognition of glycan probes in the glycan microarrays is reported in Source Data File 1 and Supplementary File 3 (MIRAGE) . The binding specificity of the anti-glycan antibodies used for studies on human vaginal epithelial cells was tested with and without specific glycan degradation by enzymatic treatments to demonstrate specificity in Supplementary Figure 6B.

Further validation of anti-GAG antibodies and Alexa Fluor 488-conjugated secondary antibodies can be found here:
https://www.abcam.com/en-us/products/primary-antibodies/chondroitin-sulfate-antibody-cs-56-ab11570#tab=datasheet
https://dshb.biology.uiowa.edu/MZ15
https://resources.amsbio.com/Datasheets/370255-S.pdf
https://www.thermofisher.com/order/genome-database/dataSheetPdf?productype=antibody&productsubtype=antibody_secondary&productId=A-21238&version=Local
https://www.thermofisher.com/order/genome-database/dataSheetPdf?producttype=antibody&productsubtype=antibody_secondary&productId=A-11001&version=Local

Eukaryotic cell lines

Policy information about cell lines and Sex and Gender in Research

Cell line source(s)	American Type Culture Collection (ATCC): VK2 E6/E7 cell line (CRL-2616)
Authentication	Cells were purchased from authenticated suppliers
Mycoplasma contamination	All cells were tested negative for mycoplasma.

Commonly misidentified lines (See ICLAC register)	No commonly misidentified cell lines were used in this study

Animals and other research organisms

Policy information about studies involving animals; ARRIVE guidelines recommended for reporting animal research, and Sex and Gender in Research

Laboratory animals	*For laboratory animals, report species, strain and age OR state that the study did not involve laboratory animals.*
Wild animals	*Provide details on animals observed in or captured in the field; report species and age where possible. Describe how animals were caught and transported and what happened to captive animals after the study (if killed, explain why and describe method; if released, say where and when) OR state that the study did not involve wild animals.*
Reporting on sex	*Indicate if findings apply to only one sex; describe whether sex was considered in study design, methods used for assigning sex. Provide data disaggregated for sex where this information has been collected in the source data as appropriate; provide overall numbers in this Reporting Summary. Please state if this information has not been collected. Report sex-based analyses where performed, justify reasons for lack of sex-based analysis.*
Field-collected samples	*For laboratory work with field-collected samples, describe all relevant parameters such as housing, maintenance, temperature, photoperiod and end-of-experiment protocol OR state that the study did not involve samples collected from the field.*
Ethics oversight	*Identify the organization(s) that approved or provided guidance on the study protocol, OR state that no ethical approval or guidance was required and explain why not.*

Note that full information on the approval of the study protocol must also be provided in the manuscript.

Plants

Seed stocks	*Report on the source of all seed stocks or other plant material used. If applicable, state the seed stock centre and catalogue number. If plant specimens were collected from the field, describe the collection location, date and sampling procedures.*
Novel plant genotypes	*Describe the methods by which all novel plant genotypes were produced. This includes those generated by transgenic approaches, gene editing, chemical/radiation-based mutagenesis and hybridization. For transgenic lines, describe the transformation method, the number of independent lines analyzed and the generation upon which experiments were performed. For gene-edited lines, describe the editor used, the endogenous sequence targeted for editing, the targeting guide RNA sequence (if applicable) and how the editor was applied.*
Authentication	*Describe any authentication procedures for each seed stock used or novel genotype generated. Describe any experiments used to assess the effect of a mutation and, where applicable, how potential secondary effects (e.g. second site T-DNA insertions, mosiacism, off-target gene editing) were examined.*

Flow Cytometry

Plots

Confirm that:

☐ The axis labels state the marker and fluorochrome used (e.g. CD4-FITC).

☒ The axis scales are clearly visible. Include numbers along axes only for bottom left plot of group (a 'group' is an analysis of identical markers).

☒ All plots are contour plots with outliers or pseudocolor plots.

☐ A numerical value for number of cells or percentage (with statistics) is provided.

Methodology

Sample preparation	Sub-confluent cultures of VK2/E6E7 cells were gently scrapped and resuspended in PBS. Chondroitinase ABC (E.C. 4.2.2.4, Sigma) and heparin lyase III (E.C.4.2.2.8, recombinant, IBEX Technologies, Montreal, Canada) were added at a concentration of 0,005 U/ml and 0,001 U/ml respectively and the cells were incubated at 37°C for 2 h. The cells were centrifuged 5 min at 300 x g and resuspended in PBS at the appropriate pH for antibody or bacteria binding analyses.<br>Single cell suspensions were stained with anti-chondroitin sulfate (CS56, Abcam), anti-keratan sulfate (MZ15, a present from Fiona Watt, Imperial, London), anti-heparan sulfate (clone 10E4, Amsbio) or biotinylated hyaluronic acid binding protein (Millipore), followed by Alexa Fluor 488-conjugated anti-mIgG (ThermoFisher) or Alexa Fluor 647-conjugated anti-mIgM antibodies (ThermoFisher) or streptavidin PE (Phycoerythrin) conjugate (Miltenyi biotech), in PBS for 30 min at 4°C.<br>For bacterial binding assays, VK2/E6E7 cells were added to bacteria suspensions to obtain a MOI=20 for all bacterial strains except for G. vaginalis at pH 4 where MOI=100 was used. Cell mixtures were gently stirred for 1 h at 4°C. Where appropriate, fluorescently labelled bacteria were incubated in PBS containing polysaccharides at 5 mg/ml for 30 min before binding assays.<br>Sub-confluent cultures of VK2/E6E7 cells were gently scrapped and resuspended in PBS. Chondroitinase ABC (E.C. 4.2.2.4, Sigma) and heparin lyase III (E.C.4.2.2.8, recombinant, IBEX Technologies, Montreal, Canada) were added at a concentration of 0,005 U/ml and 0,001 U/ml respectively and the cells were incubated at 37°C for 2 h. The cells were centrifuged 5 min at 300 x g and resuspended in PBS at the appropriate pH for antibody or bacteria binding analyses.

	Single cell suspensions were stained with anti-chondroitin sulfate (CS56, Abcam), anti-keratan sulfate (MZ15, a present from Fiona Watt, Imperial, London), anti-heparan sulfate (clone 10E4, Amsbio) or biotinylated hyaluronic acid binding protein (Millipore), followed by Alexa Fluor 488-conjugated anti-mIgG (ThermoFisher) or Alexa Fluor 647-conjugated anti-mIgM antibodies (ThermoFisher) or streptavidin PE (Phycoerythrin) conjugate (Miltenyi biotech), in PBS for 30 min at 4°C.<br>For bacterial binding assays, VK2/E6E7 cells were added to bacteria suspensions to obtain a MOI=20 for all bacterial strains except for G. vaginalis at pH 4 where MOI=100 was used. Cell mixtures were gently stirred for 1 h at 4°C. Where appropriate, fluorescently labelled bacteria were incubated in PBS containing polysaccharides at 5 mg/ml for 30 min before binding assays.
Instrument	Cells were analyzed using a FACSCalibur flow cytometer (Becton Dickinson)
Software	The data were acquired and analysed using the CellQuest software (BD Biosciences).
Cell population abundance	A homogenous cell line was used therefore all the cells were considered as relevant.
Gating strategy	The single cell population was gated based on FFS and SSC and analysed in all the experiments.

☒ Tick this box to confirm that a figure exemplifying the gating strategy is provided in the Supplementary Information.
