# Supplementary material for: Identification and characterisation of vaginal bacteria-glycan interactions implicated in reproductive tract health and pregnancy outcomes
Source: Nat Commun. 2025 Jun 5;16:5207. doi: 10.1038/s41467-025-60404-1 (PMC12137855; doi:10.1038/s41467-025-60404-1)
Supplement: Supplementary file 9 — Source Data [file 41467_2025_60404_MOESM9_ESM.zip › Source Data File 1.pdf]

Source Data File 1: Analytical results for newly prepared glycan probes.

Table SD1-1. MS Analysis of PTPA Probes.

Table SD1-2. MS Analysis of AEAB Probes.

Figure SD1-1. ESI-MS analysis of extended Ser-linked mucin Core 3.

Figure SD1-2. HPLC purification and MALDI-MS analysis of PTPA probes.

Figure SD1-3. HPLC and MS analysis of AEAB probes.

Figure SD1-4. Strong anion-exchange chromatography of GAG oligosaccharide AEAB probes.

Figure SD1-5. HPLC and ESI-MS analysis of sialyl TF antigens.

Figure SD1-6. HSQC NMR spectra of selected human milk oligosaccharides.

Figure SD1-7. HPLC and ESI-MS analysis of N-glycans released from bovine fetuin.

Table SD1-1: MS Analysis of PTPA Probes.

Index	ID	Probe Name	Mol Mass	Found
19	3151	3'Sia Hexa T2(Lex)-T1-Lac-PTPA	1823.73	1822.8 $[M-H]^-$
20	3152	3'Sia Octa-T2(Lex)-T2-T2-Lac-PTPA	2188.86	2187.7 $[M-H]^-$
21	3153	3'Sia Deca-T2-T2(Lex)-T2-T2-Lac-PTPA	2554.00	2552.9 $[M-H]^-$
22	3154	3'Sia Deca-T2-T2(Lex)-T2(Lex)-T2-Lac-PTPA	2700.05	2698.9 $[M-H]^-$
27	3132	Hexa-T1-T2-Lac-PTPA	1386.58	1387.5 $[M+H]^+$
28	3136	Hexa-T1-T2(Lex)-Lac-PTPA	1532.64	1555.5 $[M+Na]^+$
30	3131	Tetra-T2-Lac-PTPA	1021.45	1022.5 $[M+H]^+$
31	3133	Hexa-T2-T1-Lac-PTPA	1386.58	1387.5 $[M+H]^+$
32	3138	Hexa-T2-T1(Lea)-Lac-PTPA	1532.64	1555.6 $[M+Na]^+$
33	3134	Hexa-T2-T2-Lac-PTPA	1386.58	1387.5 $[M+H]^+$
34	3135	Octa-T2-T2-T2-Lac -PTPA	1751.71	1752.5 $[M+H]^+$
35	3143	Octa-T2-T2 (Lex)-T2-Lac-PTPA	1897.77	1920.8 $[M+Na]^+$
36	3147	Octa-T2-T2(Lex)-T2(Lex)-Lac-PTPA	2043.83	2066.8 $[M+Na]^+$
37	3144	Octa-T2-T2-T2(Lex)-Lac-PTPA	1897.77	1920.8 $[M+Na]^+$
38	3149	Deca-T2-T2(Lex)-T2-T2-Lac-PTPA	2262.89	2263.9 $[M+H]^+$
39	3150	Deca-T2-T2(Lex)-T2(Lex)-T2-Lac-PTPA	2408.96	2431.9 $[M+Na]^+$
41	3120	A Hexa T1-T2-Lac-PTPA	1735.72	1736.6 $[M+H]^+$
43	3117	A Tetra T2-Lac-PTPA	1370.58	1393.7 $[M+Na]^+$
44	3123	A Hexa T2-T1-Lac-PTPA	1735.72	1736.6 $[M+H]^+$
48	3121	B Hexa T1-T2-Lac-PTPA	1694.69	1695.7 $[M+H]^+$
50	3118	B Tetra T2-Lac-PTPA	1329.56	1352.7 $[M+Na]^+$
51	3124	B Hexa T2-T1-Lac-PTPA	1694.69	1695.6 $[M+H]^+$
54	3119	H Hexa T1-T2-Lac-PTPA	1532.64	1533.6 $[M+H]^+$
56	3116	H Tetra T2-Lac-PTPA	1167.50	1190.6 $[M+Na]^+$
57	3122	H Hexa T2-T1-Lac-PTPA	1532.64	1533.5 $[M+H]^+$
61	3125	Hexa T1(Leb)-T2-Lac-PTPA	1678.69	1701.7 $[M+Na]^+$
62	3126	A Hexa T1(Leb)-T2-Lac-PTPA	1881.77	1904.7 $[M+Na]^+$
63	3127	B Hexa T1(Leb)-T2-Lac-PTPA	1840.75	1863.7 $[M+Na]^+$
65	3128	Hexa T2(Ley)-T1-Lac-PTPA	1678.69	1701.7 $[M+Na]^+$
66	3129	A Hexa T2(Ley)-T1-Lac-PTPA	1881.77	1904.8 $[M+Na]^+$
67	3130	B Hexa T2(Ley)-T1-Lac-PTPA	1840.75	1863.7 $[M+Na]^+$
69	3137	Hexa T1(Lea)-T2-Lac-PTPA	1532.64	1555.5 $[M+Na]^+$
70	3140	Hexa T1(Lea)-T2(Lex)-Lac-PTPA	1678.69	1701.7 $[M+Na]^+$
72	3139	Hexa T2(Lex)-T1-Lac-PTPA	1532.64	1555.6 $[M+Na]^+$
73	3141	Hexa T2(Lex)-T1(Lea)-Lac-PTPA	1678.69	1701.7 $[M+Na]^+$
74	3142	Octa-T2(Lex)-T2-T2-Lac-PTPA	1897.77	1920.8 $[M+Na]^+$
75	3145	Octa-T2(Lex)-T2(Lex)-T2-Lac-PTPA	2043.83	2066.8 $[M+Na]^+$
76	3146	Octa-T2(Lex)-T2-T2(Lex)-Lac-PTPA	2043.83	2066.8 $[M+Na]^+$
77	3148	Octa-T2(Lex)-T2(Lex)-T2(Lex)-Lac-PTPA	2189.87	2212.7 $[M+Na]^+$

Table SD1-2: MS Analysis of AEAB Probes.

Probe Index	Probe ID	Probe Name	Found	Assignment	Calc'd	Theor
13	3210	N-Glycan A2 (Fetuin)-AEAB	1192.1	[M-2H]2-	2386.2	2385.89
14	3211	N-Glycan A3 (Fetuin)-AEAB	1520.7	[M-2H]2-	3043.4	3042.12
15	3212	N-Glycan A3 (Fetuin)-AEAB	1520.7	[M-2H]2-	3043.4	3042.12
16	3356	LSTa-AEAB	1160.5	[M-H]-	1161.5	1161.45
17	3359	LSTd-AEAB	1160.5	[M-H]-	1161.5	1161.45
18	3360	SA2,3-Octa(T2)-AEAB	944.8	[M-2H]2-	1891.6	1891.72
23	2333	SSEA4-Hexa-AEAB (03/22)	1322.4	[M-H]-	1323.4	1323.51
24	3357	LSTb-AEAB	1160.5	[M-H]-	1161.5	1161.45
25	3358	LSTc-AEAB	1160.5	[M-H]-	1161.5	1161.45
26	3361	SA2,6-Octa(T2)-AEAB	944.8	[M-2H]2-	1891.6	1891.72
29	3326	LNnT-AEAB(05/23)	871.5	[M+H]+	870.5	870.36
40	3158	A-T1-Hexa-AEAB	1220.6	[M+H]+	1219.6	1219.50
42	3159	A-T2-Hexa-AEAB	1220.6	[M+H]+	1219.6	1219.50
45	3160	A-T4-Penta-AEAB	1058.6	[M+H]+	1057.6	1057.44
46	2340	Globo A-Hepta-AEAB (03/22)	1380.6	[M-H]-	1381.6	1381.55
47	3161	B-T1-Hexa-AEAB	1179.6	[M+H]+	1178.6	1178.47
49	3162	B-T2-Hexa-AEAB	1179.6	[M+H]+	1178.6	1178.47
52	2341	Globo B-Hepta-AEAB (03/22)	1339.4	[M-H]-	1340.4	1340.52
53	3330	LNFPI-AEAB[09/22]	1017.4	[M+H]+	1016.4	1016.42
55	3164	H-T2-(LNnFPI)-AEAB	1017.5	[M+H]+	1016.5	1016.42
58	2342	Globo H-Hexa-AEAB (03/22)	1177.5	[M-H]-	1178.5	1178.47
59	3336	Leb-Penta-AEAB	1001.5	[M+H]+	1000.5	1000.42
60	3168	Leb-Hexa-(LNDFHI)-AEAB	1163.6	[M+H]+	1162.6	1162.48
64	3169	Ley-Penta-AEAB	1001.5	[M+H]+	1000.5	1000.42
68	3165	Lea-Penta-(LNFPII)-AEAB	1017.5	[M+H]+	1016.5	1016.42
71	3334	LNFPIII-AEAB [09/22]	1017.5	[M+H]+	1016.5	1016.42
78	2751	HMO Tang HP1-AEAB	1382.5	[M+H]+	1381.5	1381.55
82	3257	MFLNH I-AEAB	1404.8	[M+Na]+	1381.8	1381.55
83	3258	DFLNH(a)-AEAB	1551.0	[M+Na]+	1528.0	1527.61
84	3255	TFiLNO-AEAB	2062.4	[M+Na]+	2039.4	2038.80
85	2752	HMO Tang HP2-AEAB	1528.5	[M+H]+	1527.5	1527.61
86	3256	TFLNH-AEAB	1697.1	[M+Na]+	1674.1	1673.67
88	3338	Chondroitin(S0) DP-10-AEAB	1028.3	[M-2H]2-	2058.6	2058.67
89	3340	Chondroitin(S0) DP-12-AEAB	1217.8	[M-2H]2-	2437.6	2437.78
90	3342	Chondroitin(S0) DP-14-Aeab	1407.4	[M-2H]2-	2816.8	2816.89
111	3193	CSA-DP6.AEAB	384.1	[M-4H]4-	1540.4	1540.32
112	3194	CSA-DP10(3S)-AEAB	573.7	[M-5H]5-	2298.8	2298.54
113	3195	CSA-DP10(5S)-AEAB	408.8	[M-6H]6-	2458.8	2458.45
114	3197	CSA-DP14(6S)-AEAB	548.5	[M-6H]6-	3297.0	3296.63
115	3198	CSB-DP6(2S)-AEAB	485.8	[M-2H]2-	1460.4	1460.36
116	3199	CSB-DP6(3S)-AEAB	384.1	[M-4H]4-	1540.4	1540.32
117	3200	CSB-DP10(4S)-AEAB	1188.3	[M-2H]2-	2378.6	2378.50
118	3201	CSB-DP10(5S)-AEAB	408.8	[M-6H]6-	2458.8	2458.45
119	3202	CSB-DP14(6S)-AEAB	548.5	[M-6H]6-	3297.0	3296.63
120	3203	CSC-DP6-AEAB	769.2	[M-2H]4-	1540.4	1540.32
121	3204	CSC-DP10-AEAB	408.8	[M-6H]6-	2458.8	2458.45
122	3205	CSC-DP14-AEAB	481.4	[M-7H]7-	3376.8	3376.59
123	3224	Hep-DP6-AEAB	786.2	[M-2H]2-	1574.4	1574.20
124	3225	Hep-DP10-AEAB	855.2	[M-3H]3-	2568.6	2568.23
125	3206	Hep-DP14-AEAB	1160.0	[M-3H]3-	3483.0	3482.30
126	3226	KS-DP4-0S-AEAB	910.5	[M-H]-	911.5	911.39
127	3228	KS-DP6-0S-AEAB	1275.7	[M-H]-	1276.5	1276.52
128	3236	KS-DP8-0S-AEAB	1640.9	[M-H]-	1641.9	1641.65
129	3227	KS-DP4-4S-AEAB	382.8	[M-3H]3-	1231.5	1231.21

Table SD1-2 (cont.): MS Analysis of AEAB Probes.

**130**	3229	KS-DP6-1S(a)-AEAB	1355.6	[M-H]-	1356.6	1356.47
**131**	3230	KS-DP6-1S(b)-AEAB	1355.6	[M-H]-	1356.6	1356.47
**132**	3231	KS-DP6-1S(c)AEAB	1355.6	[M-H]-	1356.6	1356.47
**133**	3232	KS-DP6-2S(a)-AEAB (L221)	717.3	[M-2H]2-	1436.6	1436.43
**134**	3233	KS-DP6-2S(b)-AEAB (L212)	717.3	[M-2H]2-	1436.6	1436.43
**135**	3234	KS-DP6-2S(c)-AEAB (L122)	717.3	[M-2H]2-	1436.6	1436.43
**136**	3238	KS(II)-DP10 (LS)-AEAB	412.5	[M-6H]6-	2487.0	2486.53
**137**	3240	KS(II)-DP14 (LS)-AEAB	470.0	[M-7H]7-	3297.0	3296.74
**138**	3241	KS(II)-DP14 (HS)-AEAB	383.1	[M-9H]9-	3456.9	3456.66
**139**	3185	HA-DP4-H-AEAB	468.7	[M-2H]2-	939.4	939.34
**140**	3186	HA-DP8-H-AEAB	564.9	[M-4H]4-	1697.7	1697.57
**141**	3187	HA-DP12-H-AEAB	490.2	[M-5H]5-	2456.0	2455.79
**142**	3188	HA-DP14-H-AEAB	471.5	[M-6H]6-	2835.0	2834.90
**143**	3189	HA-DP11-L-AEAB	569	[M-4H]4-	2280.0	2279.76
**144**	3190	HA-DP12-L-AEAB	486.6	[M-5H]5-	2438.0	2437.78
**145**	3191	HA-DP13-L-AEAB	530.8	[M-5H]5-	2659.0	2658.87
**146**	3192	HA-DP14-L-AEAB	468.5	[M-6H]6-	2817.0	2816.89

#178 (Gal-Core 3-Ser)

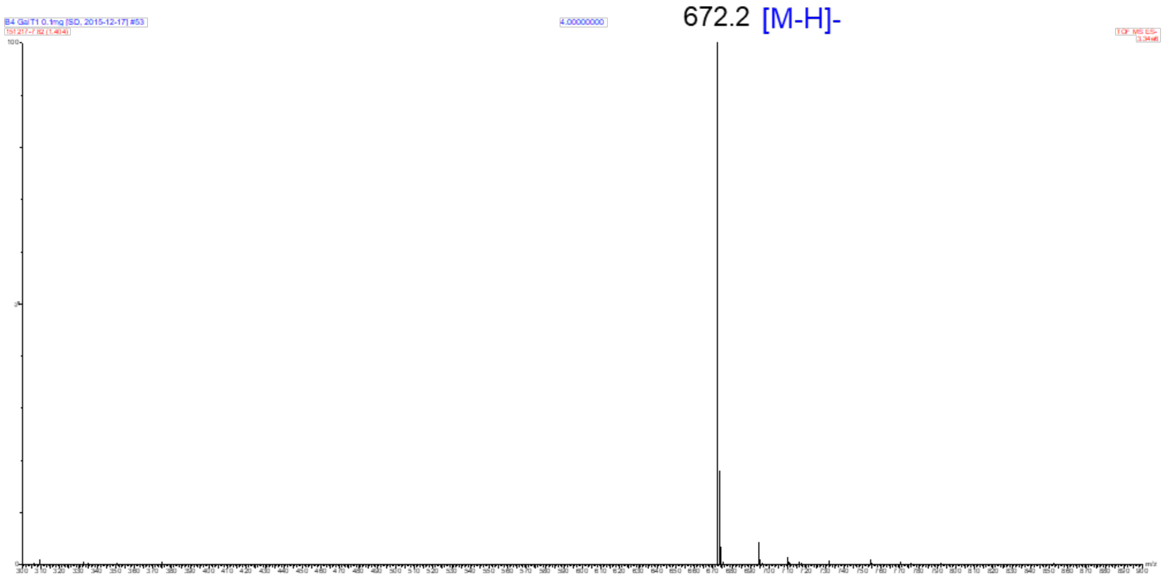

#179 (G(3S)-Core 3-Ser)

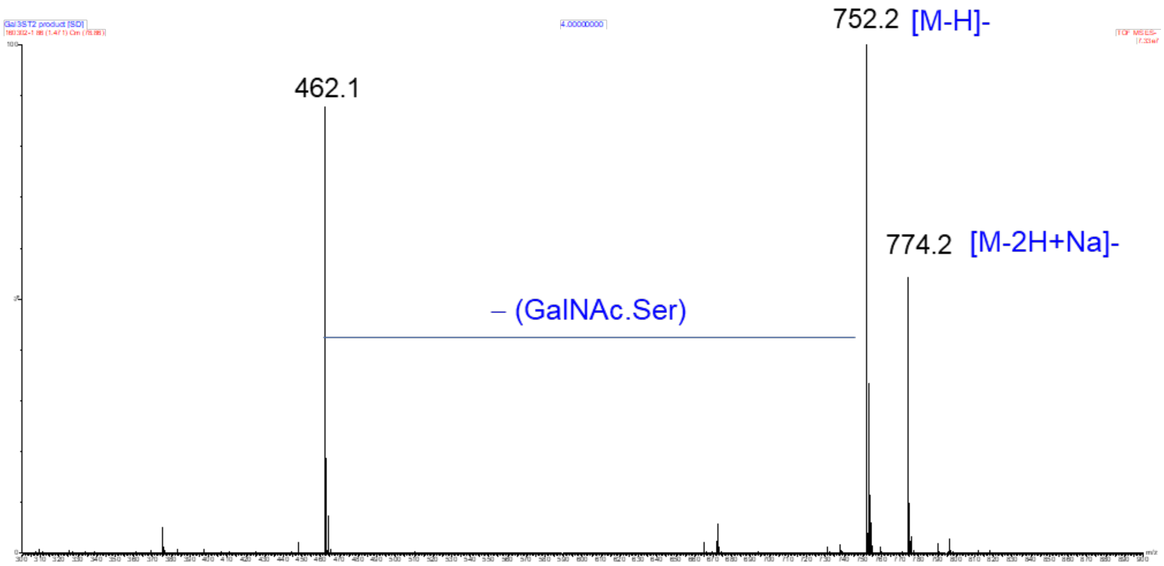

Figure SD1-1. ESI-MS analysis of extended Ser-linked mucin Core 3.

#180 (GalNAc-G(3S)-Core 3-Ser)

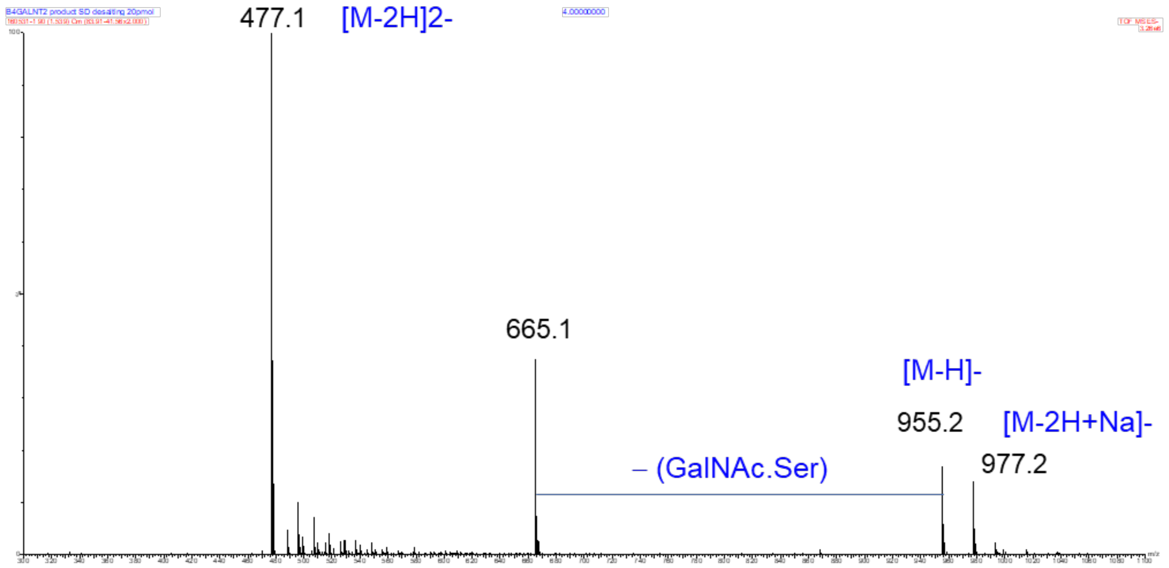

#181 (GSC967-Ser)

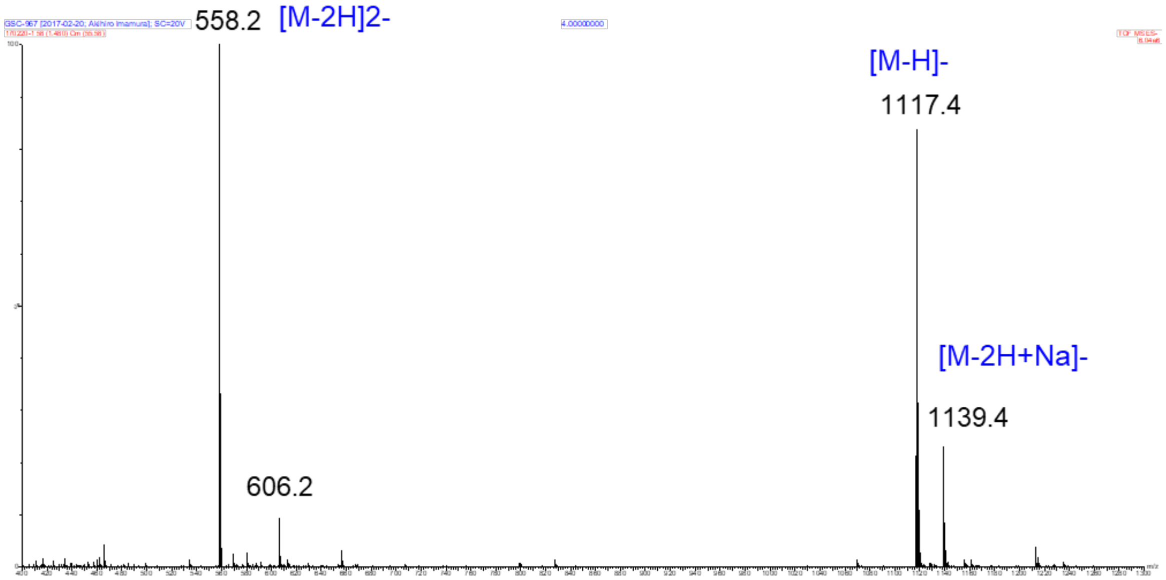

Figure SD1-1 cont. ESI-MS analysis of extended Ser-linked mucin Core 3.

(a) Probe #20 (3'Sia Octa-T2(Lex)-T2-T2-Lac-PTPA)

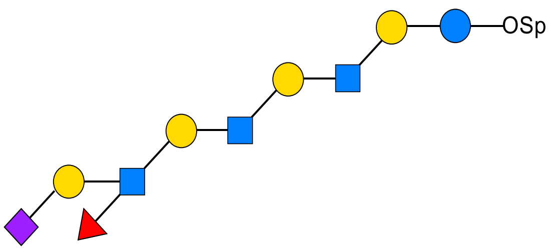

HPLC Purification of PTPA conjugation Product

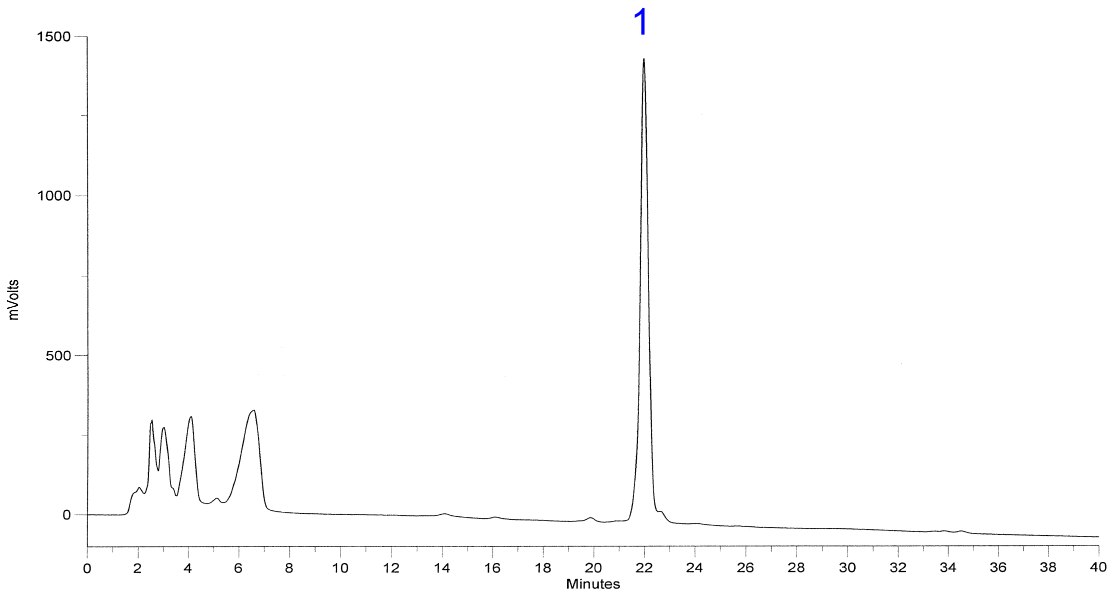

MALDI-MS analysis of fraction HPLC-1

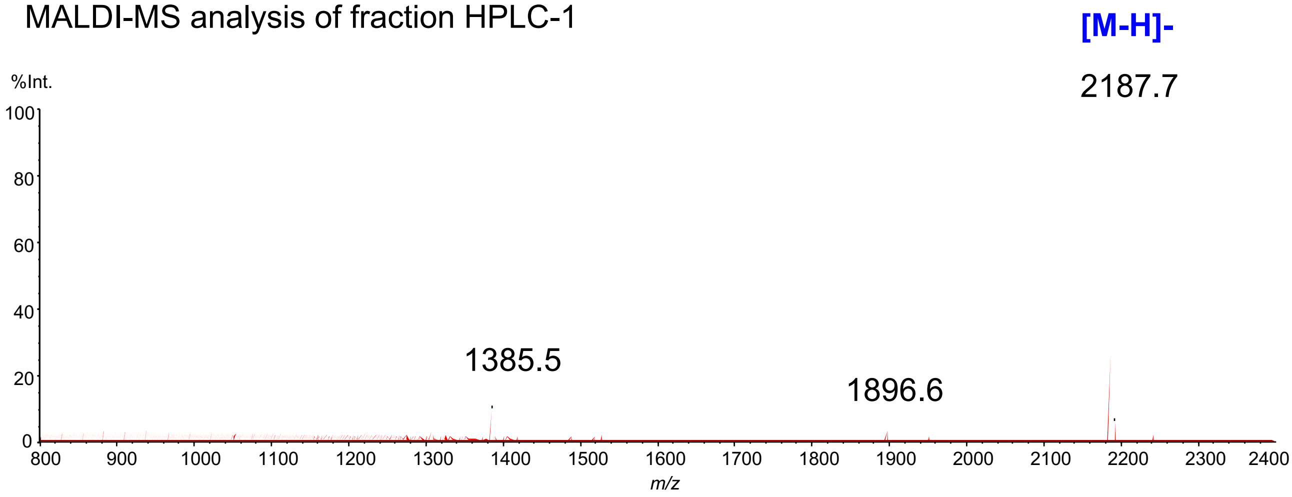

Figure SD1-2a. HPLC purification and MALDI-MS analysis of PTPA probe # 20.

(b) Probe #22 (3'Sia Deca-T2-T2(Lex)-T2-T2-Lac-PTPA)

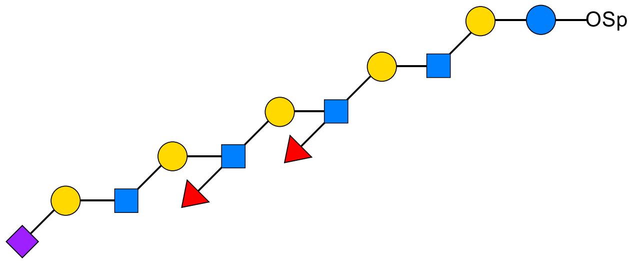

HPLC Purification of PTPA conjugation Product

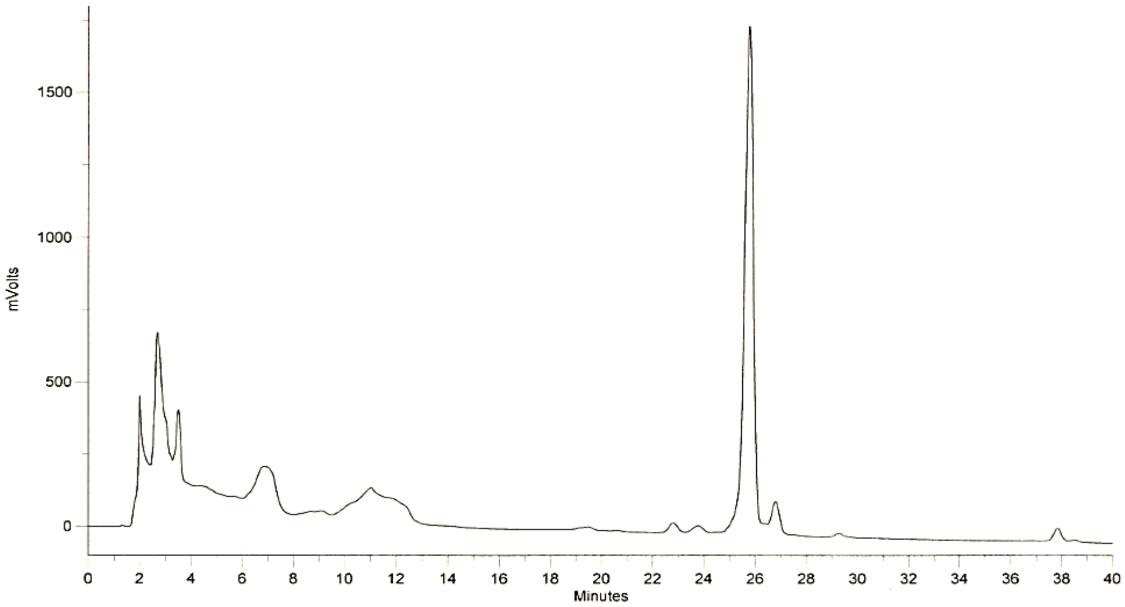

MS analysis of HPLC Fraction 1

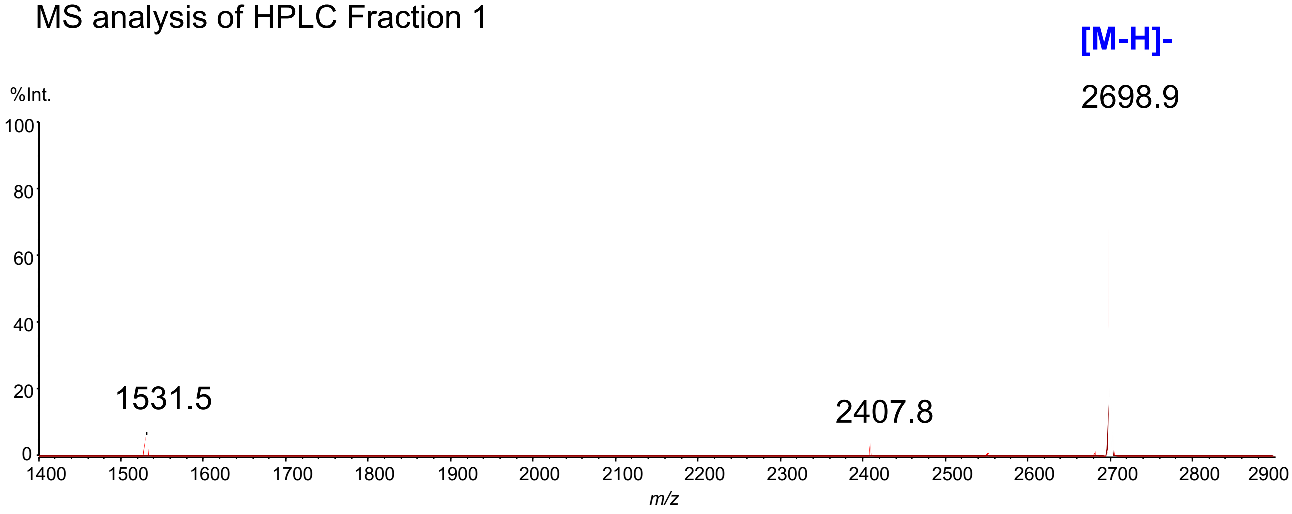

Figure SD1-2b. HPLC purification and MALDI-MS analysis of PTPA probe # 22.

Probe #46 **(Globo-A-Hepta-AEAB)**

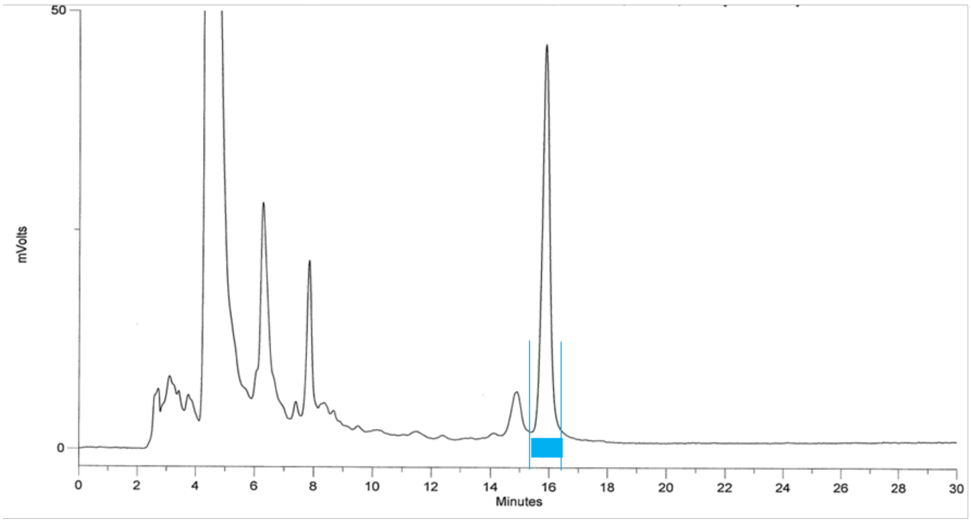

Probe #52 **(Globo-H-Hexa-AEAB)**

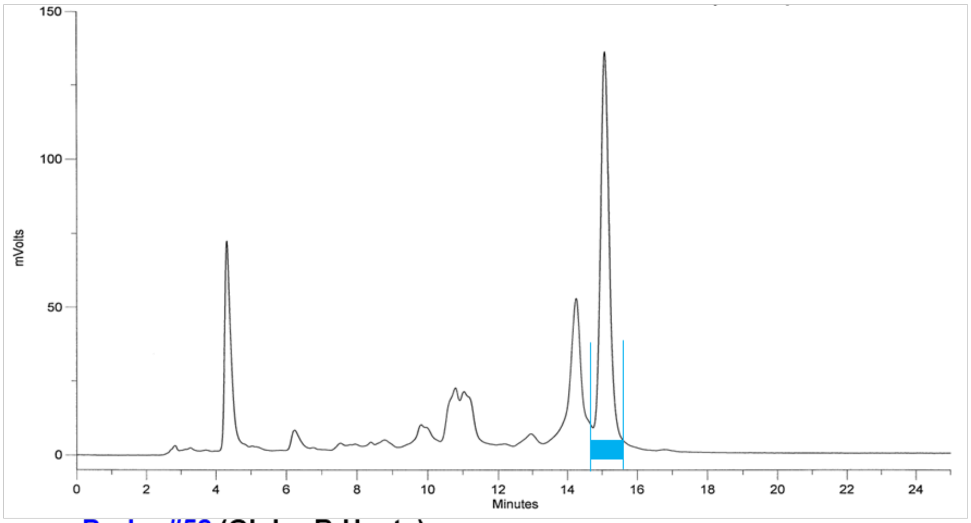

Probe #58 **(Globo-B-Hepta)**

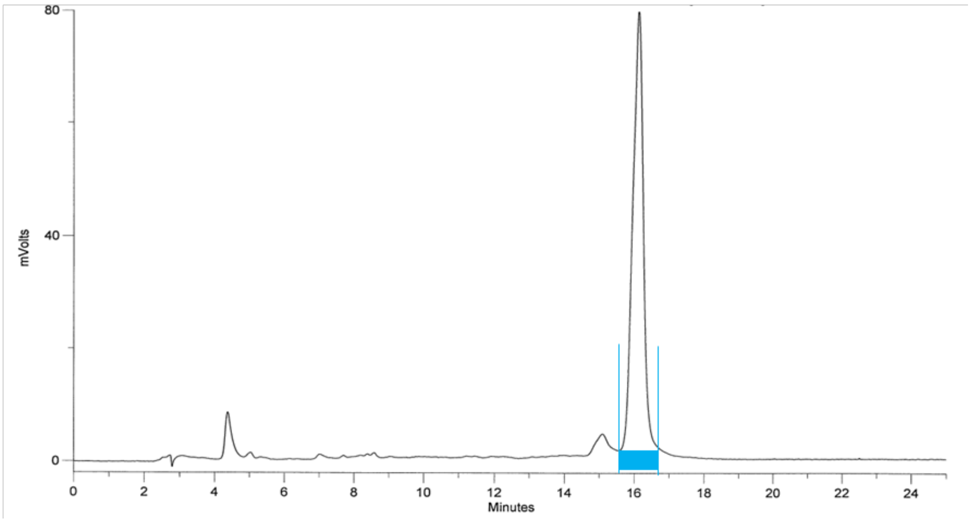

Figure SD1-3a. Final purification of AEAB probes by HPLC.

Probe #40 (A-T1-Hexa-AEAB)

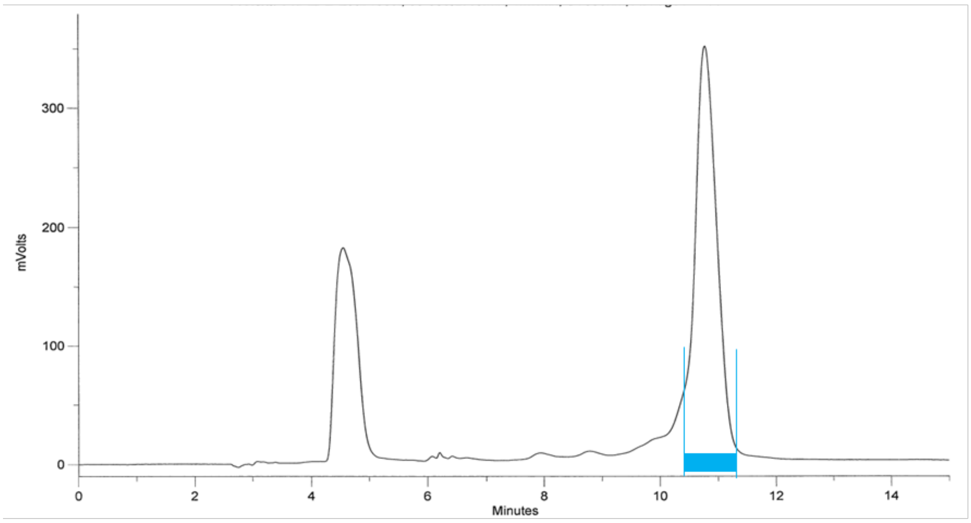

Probe #47 (B-T1-Hexa-AEAB)

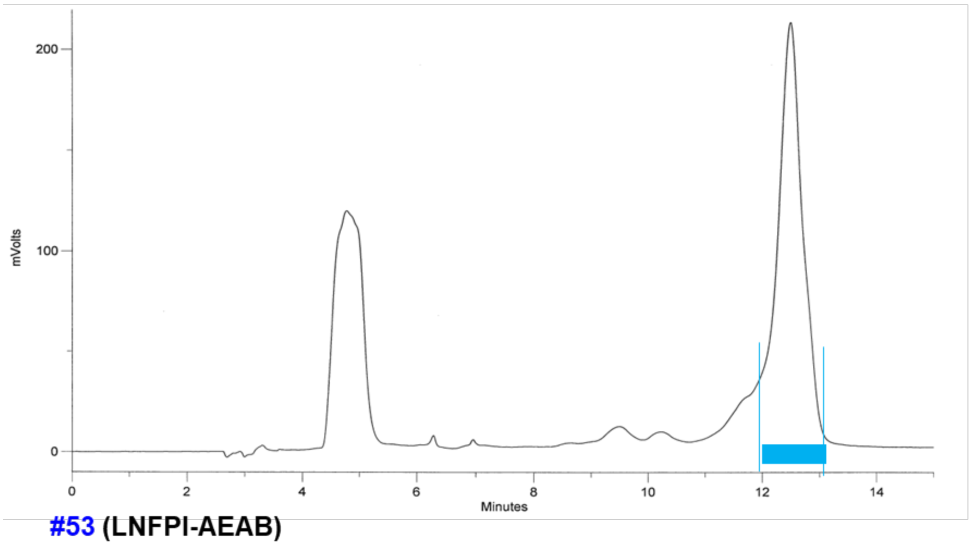

#53 (LNFPI-AEAB)

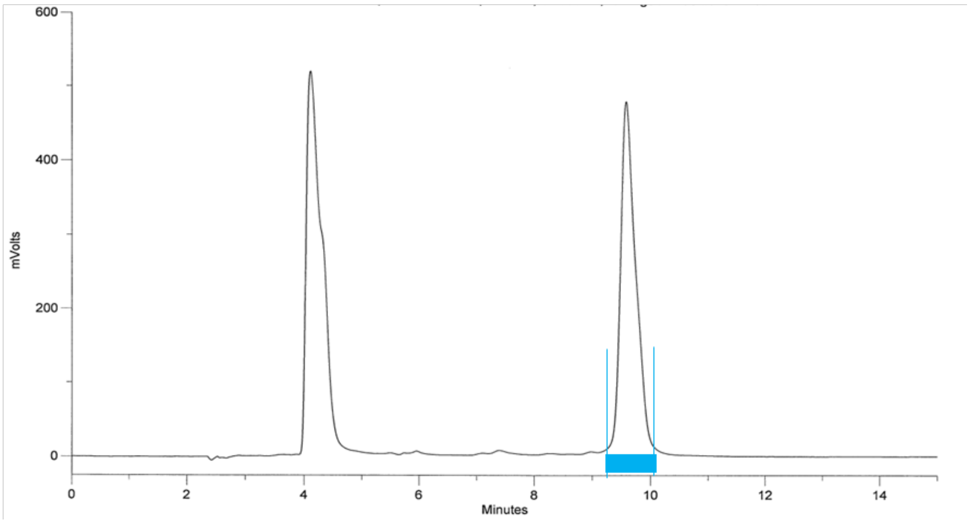

Figure SD1-3a cont. Final purification of AEAB probes by HPLC.

Probe #68 (LNFPII-AEAB)

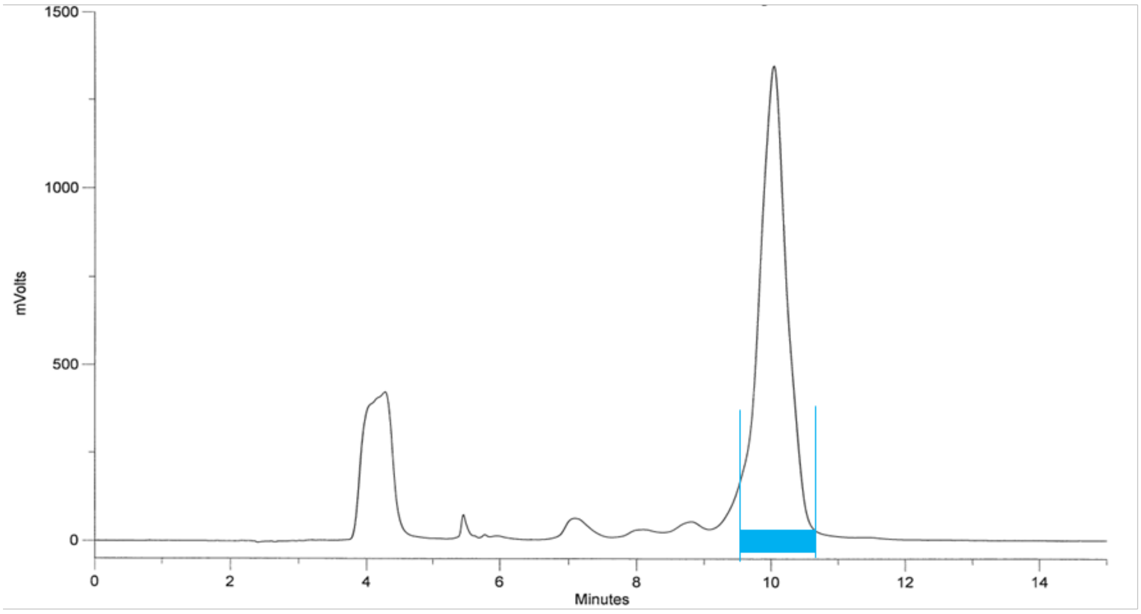

Probe #71 (LNFPIII-AEAB)

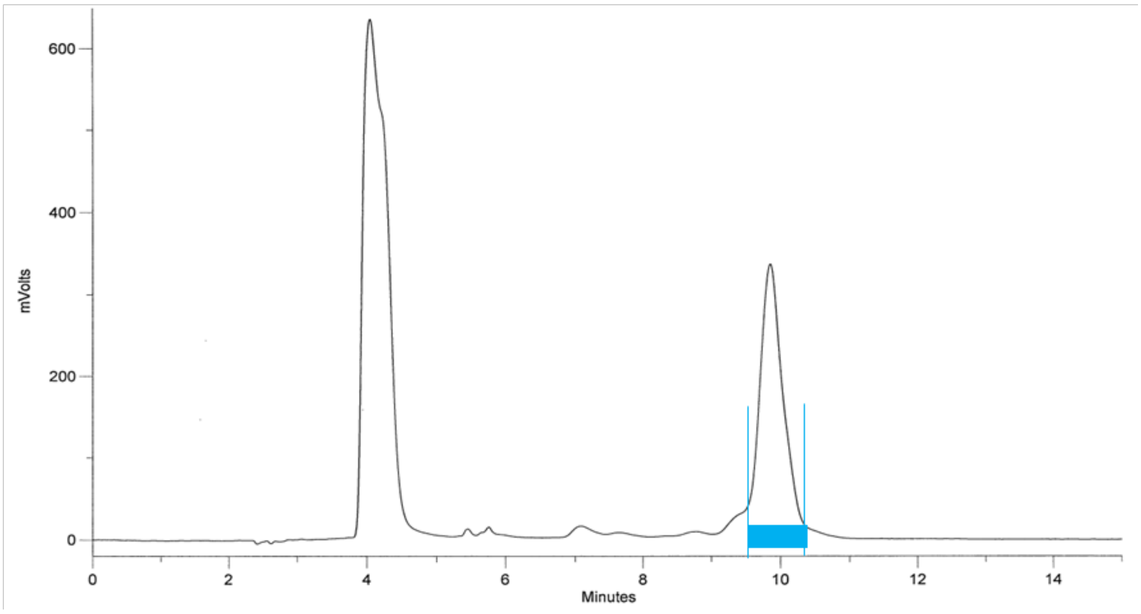

Figure SD1-3a cont. Final purification of AEAB probes by HPLC.

Probe #140 **(HA-DP8-H)**

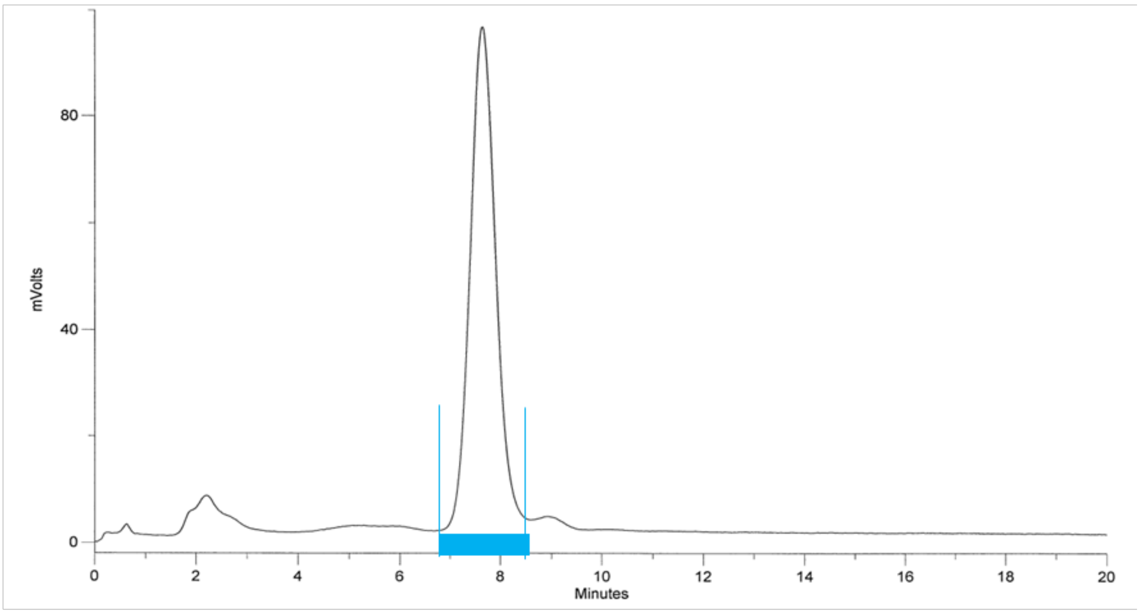

Probe #142 **(HA-DP14-H)**

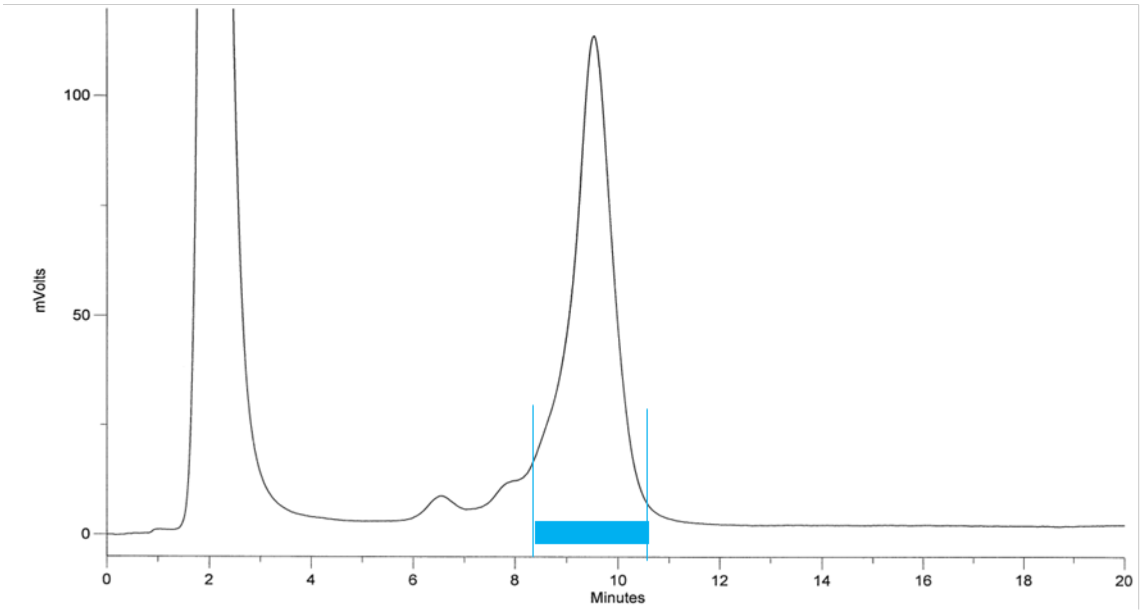

Figure SD1-3a cont. Final purification of AEAB probes by HPLC.

Probe #53 (LNFPI-AEAB)

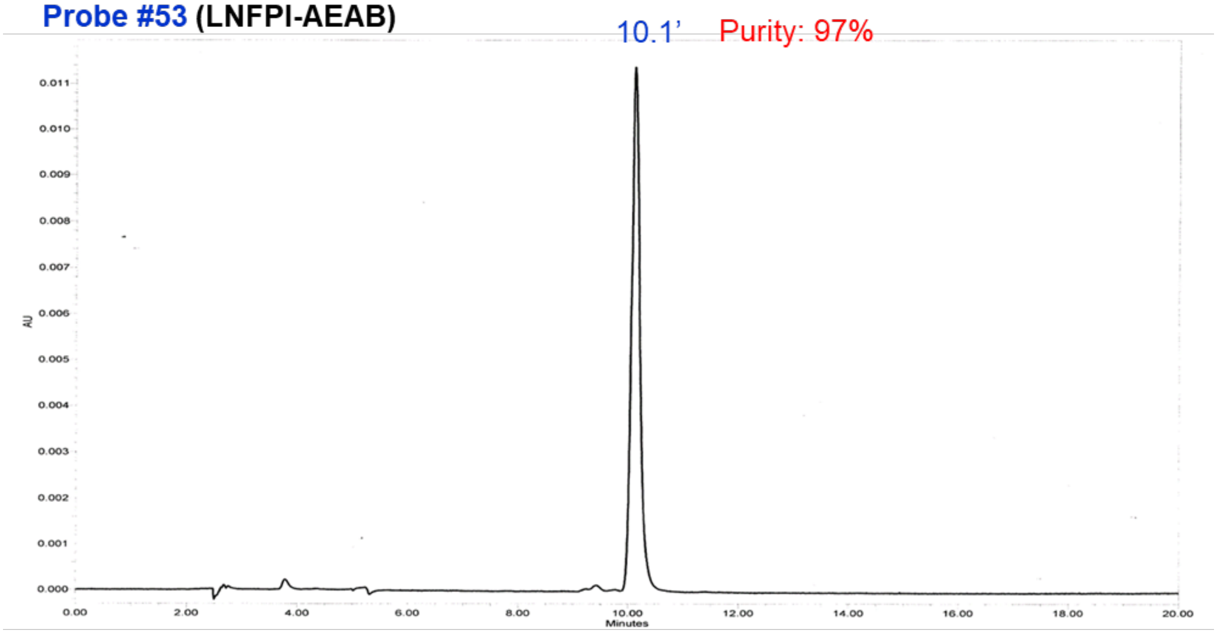

Probe #55 (LNnFPI-AEAB)

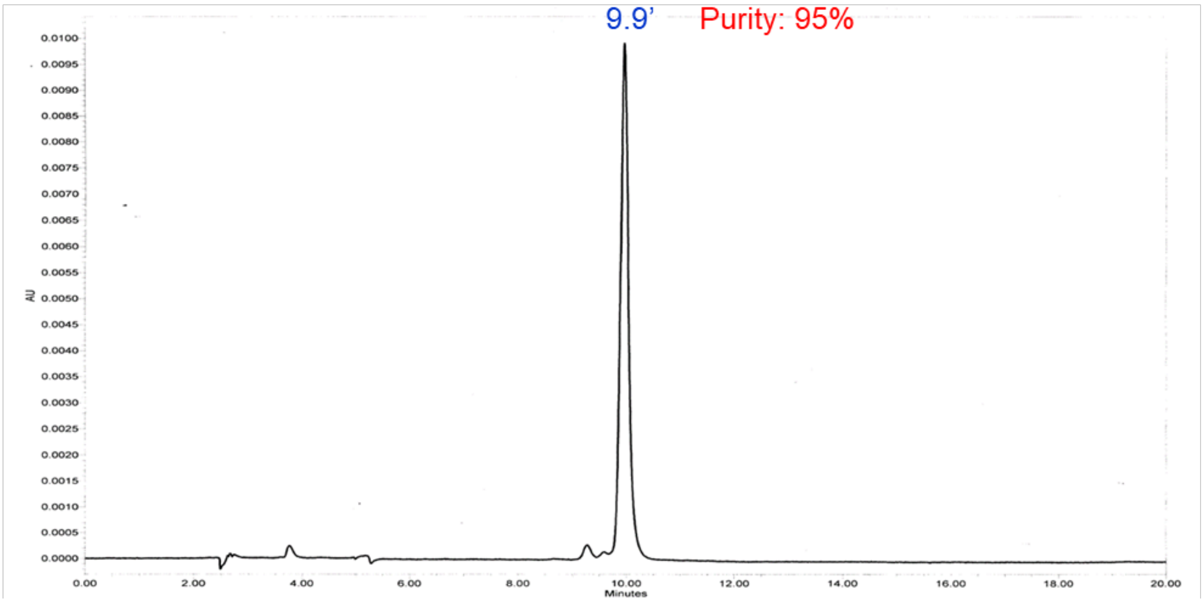

Figure SD1-3b. Purity analysis of AEAB probes by HPLC.

Probe #68 **(LNFPII-AEAB)**

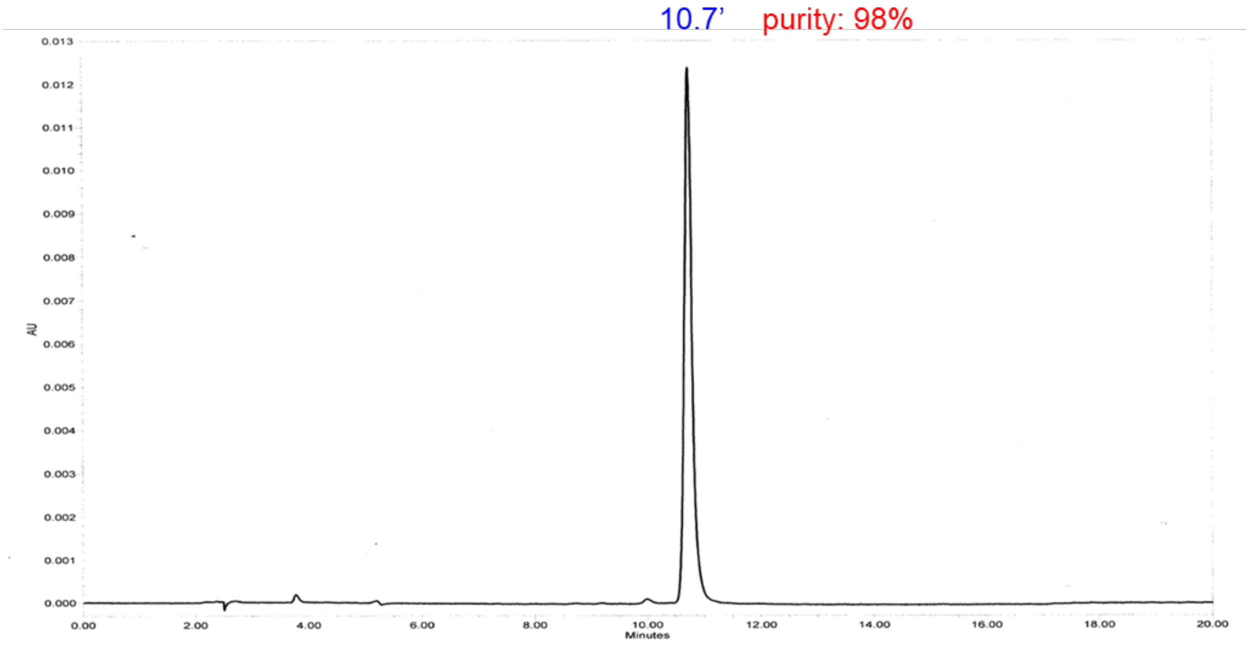

Probe #71 **(LNFPIII-AEAB)**

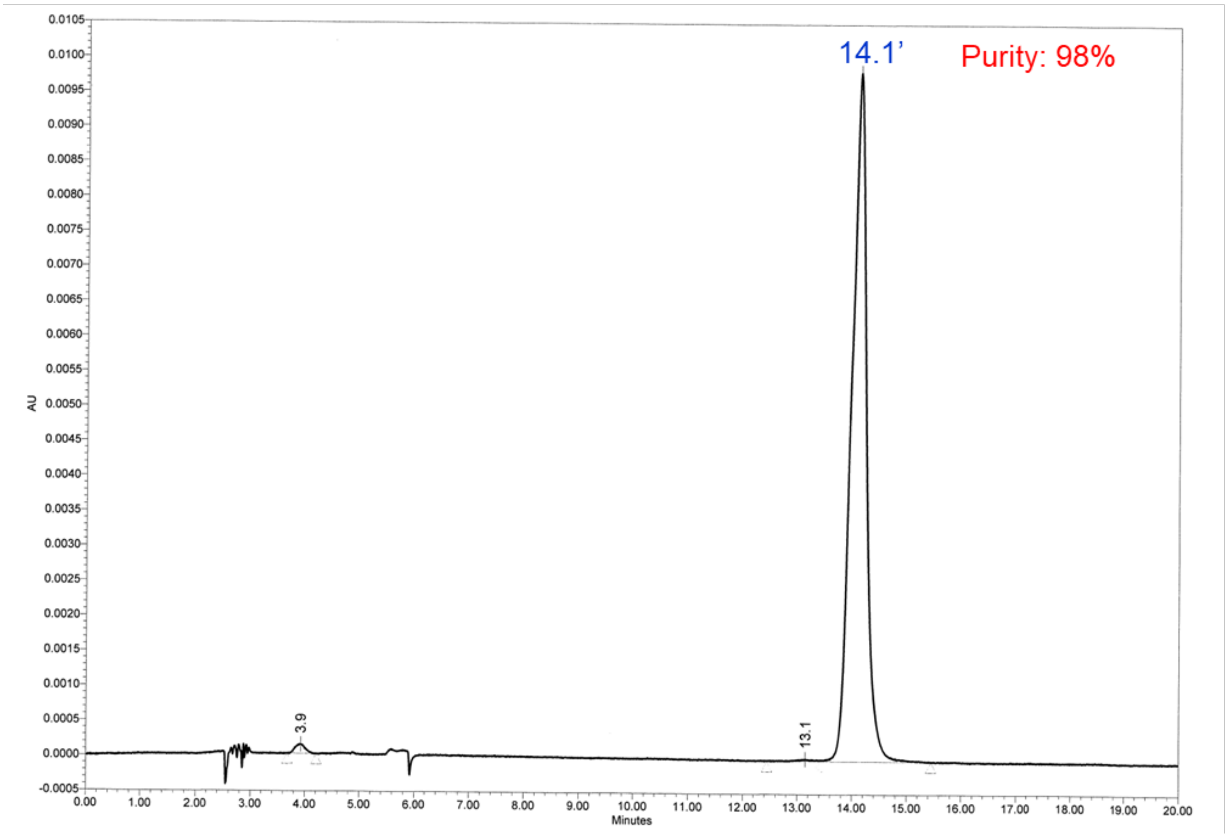

Figure SD1-3b cont. Purity analysis of AEAB probes by HPLC.

Probe #59 **(Lewis b-Penta-AEAB)**

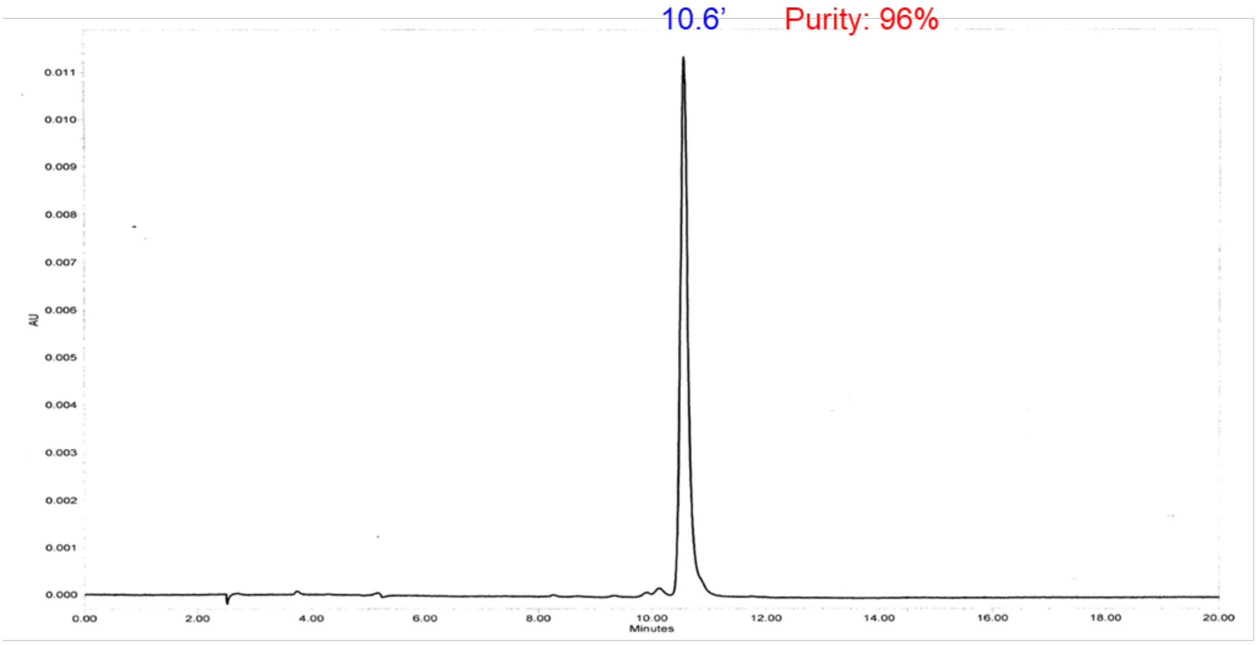

Probe #60 **(LNDFHI-AEAB)**

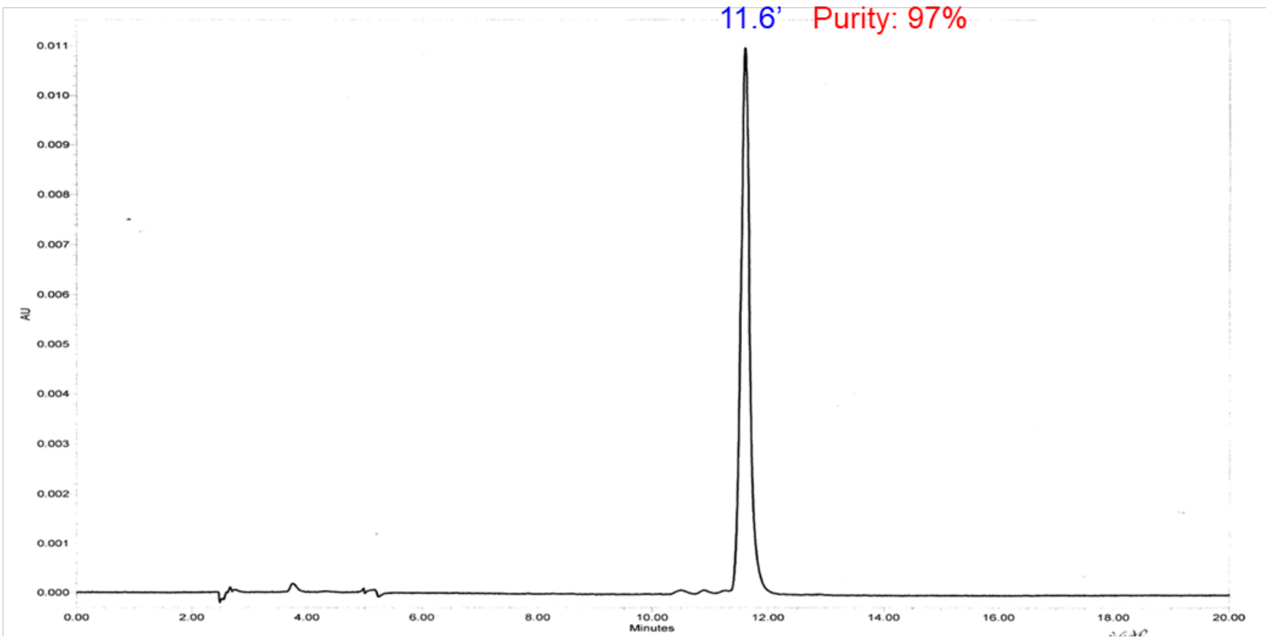

Figure SD1-3b cont. Purity analysis of AEAB probes by HPLC.

Probe #29 **(LNnT-AEAB)**

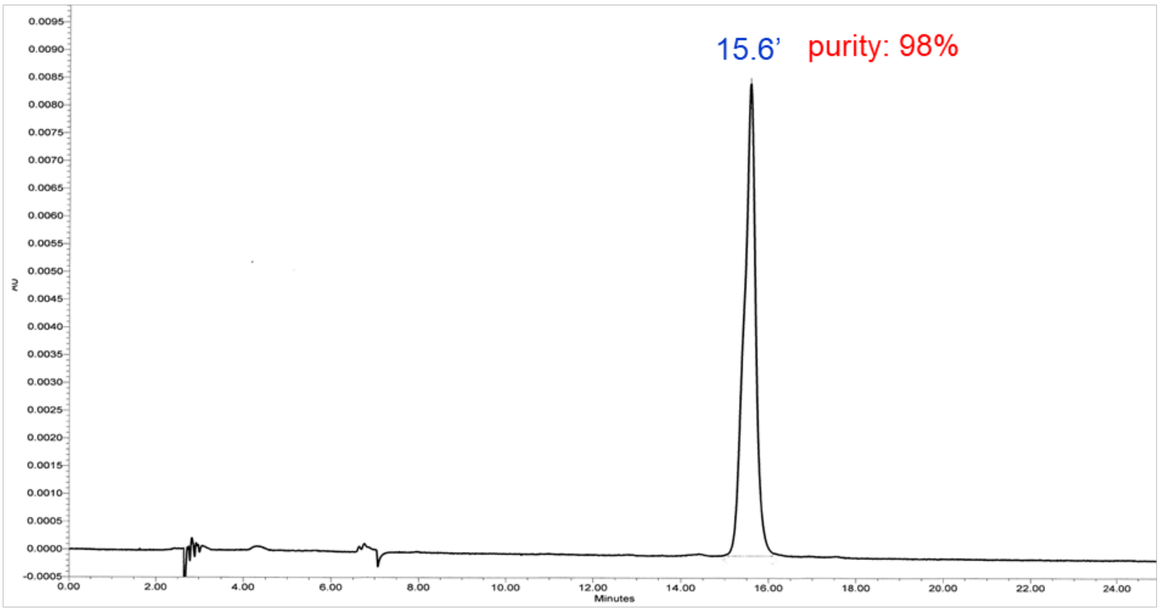

Probe #64 **(Ley-Penta-AEAB)**

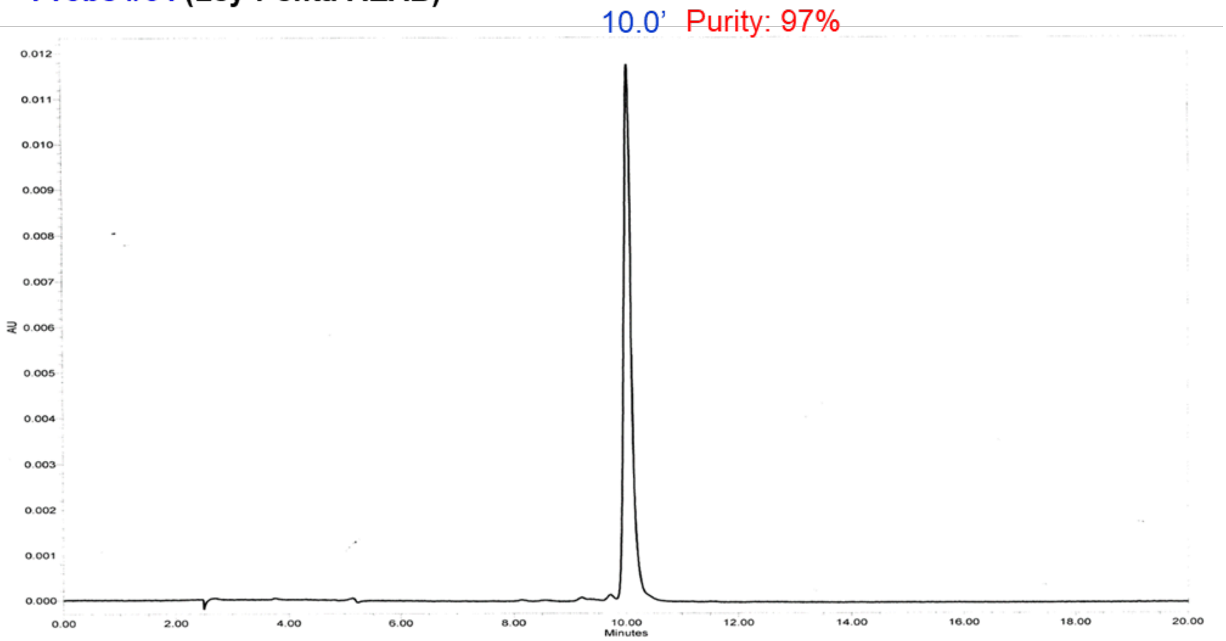

Figure SD1-3b cont. Purity analysis of AEAB probes by HPLC.

Probe #68 (LNFPII-AEAB)

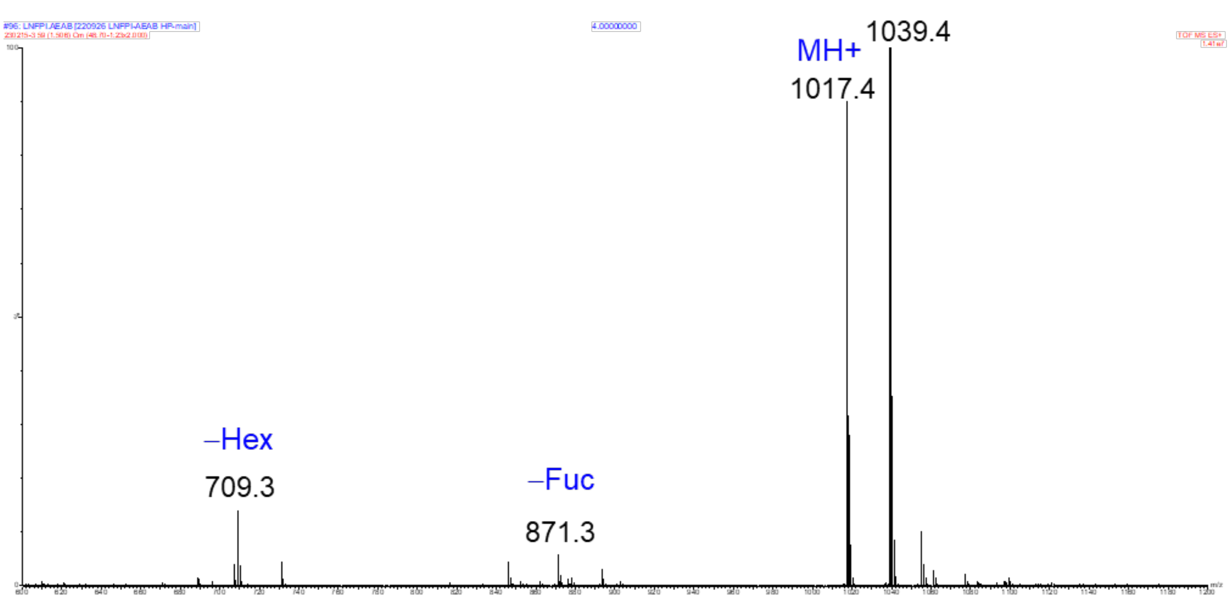

Probe #71 (LNFPIII-AEAB)

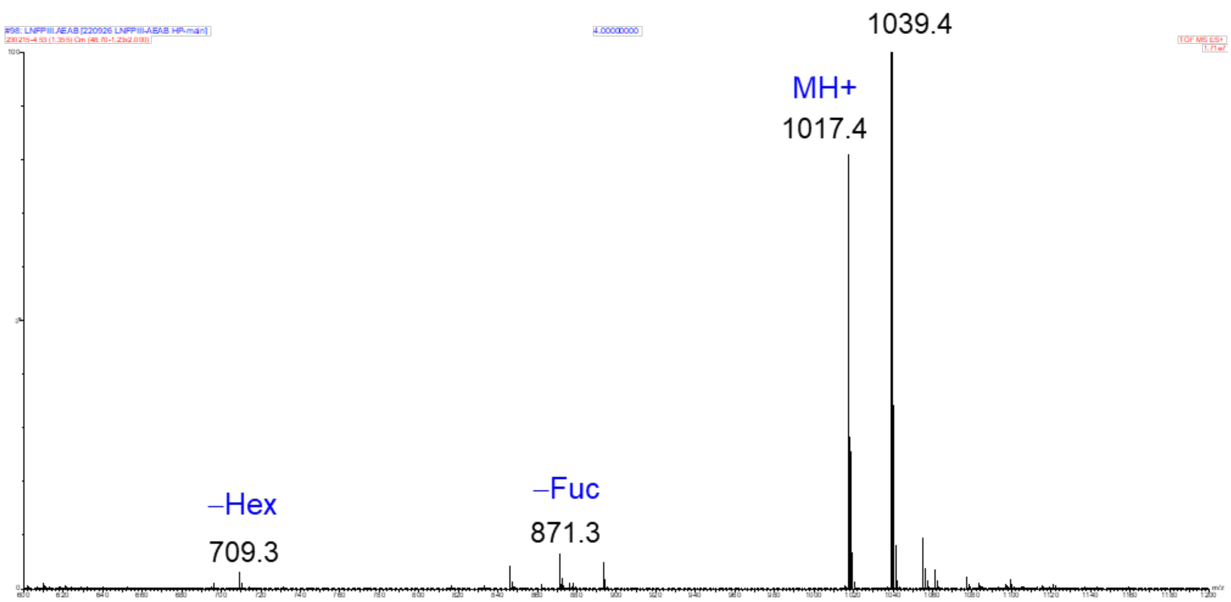

Figure SD1-3c. Mass spectra of purified AEAB probes.

#29 **(LNnT-AEAB)**

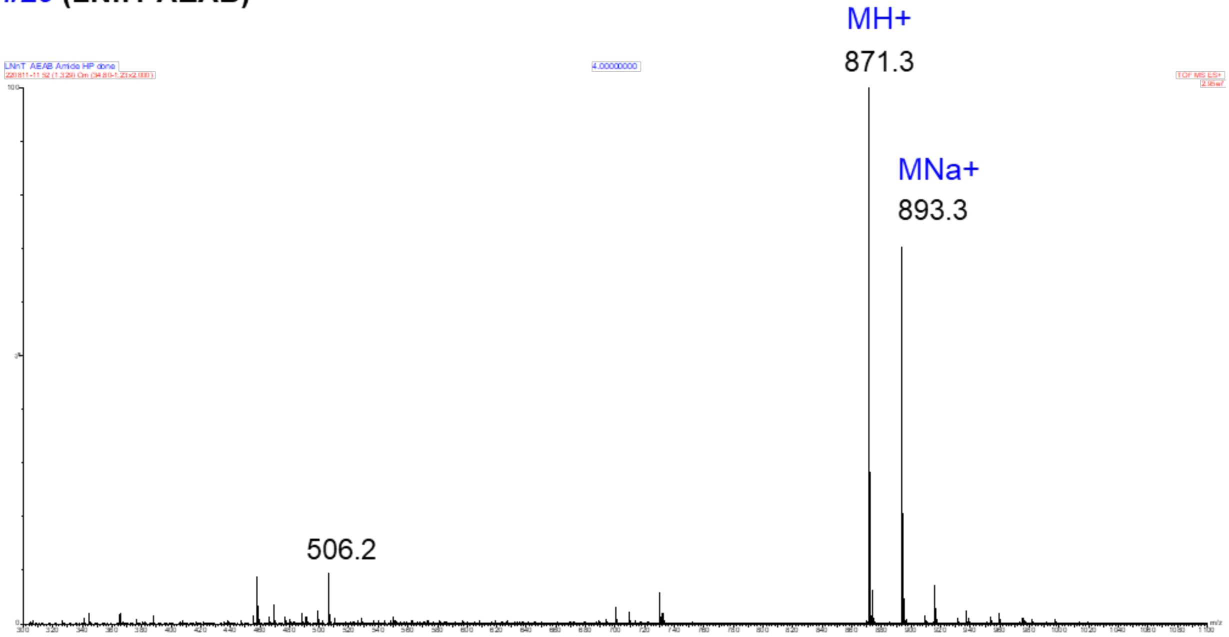

Figure SD1-3c cont. Mass spectra of purified AEAB probes.

Probe #114 (CSA-DP14-AEAB)

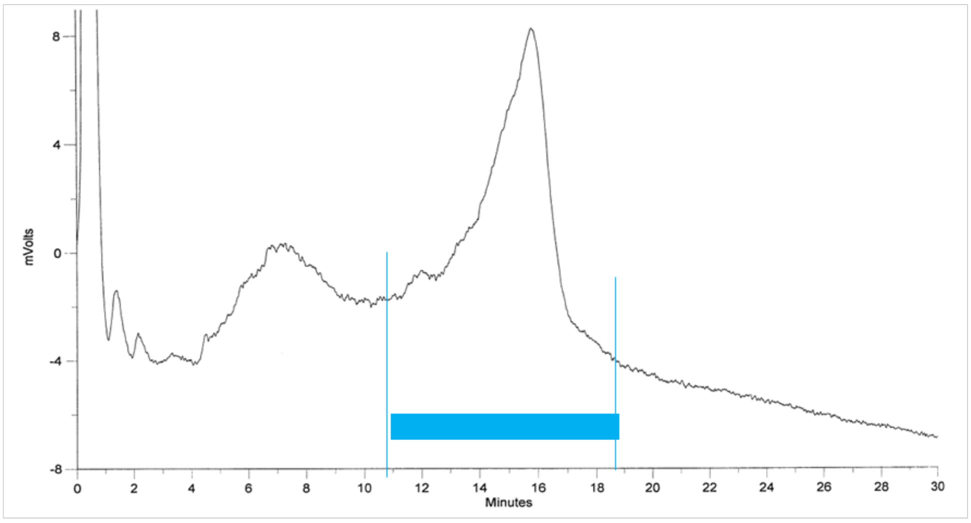

Probe #119 (CSB-DP14-AEAB)

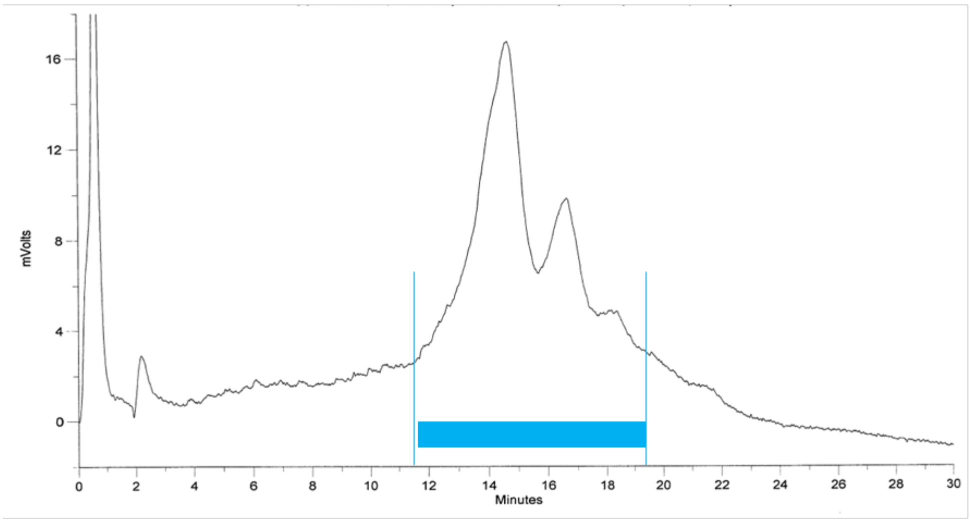

Probe #122 (CSC-14-AEAB)

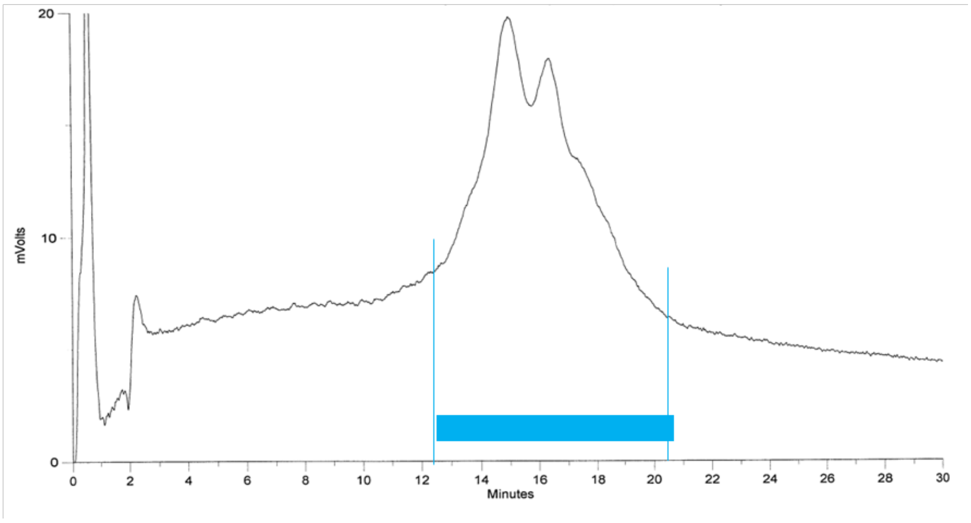

Figure SD1-4. Final step of purification of GAG oligosaccharide AEAB probes by strong anion-exchange chromatography.

Probe #125 **(Heparin-DP14-AEAB)**

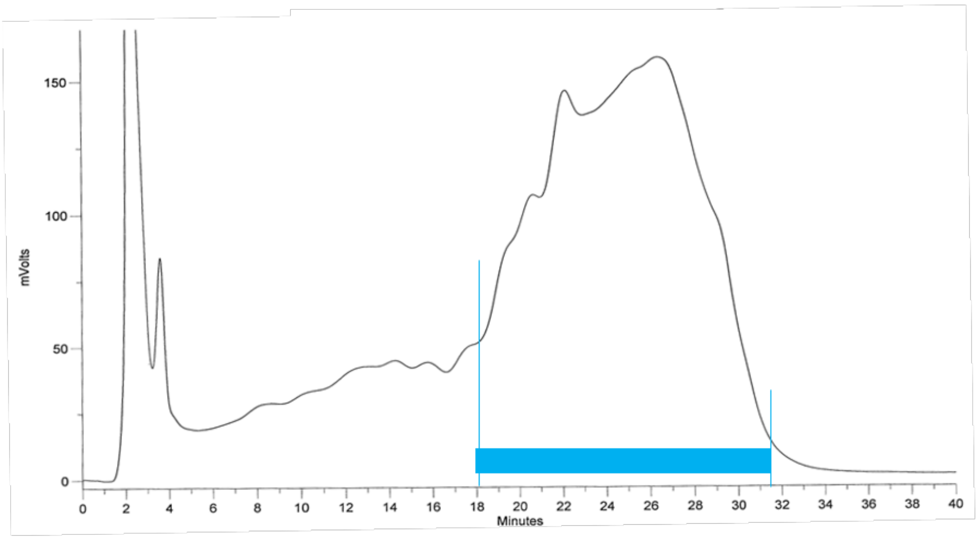

Figure SD1-4 cont. Final step of purification of GAG oligosaccharide AEAB probes by strong anion-exchange chromatography.

HPLC profile of a crude STF antigen fraction (F26) from human urine.

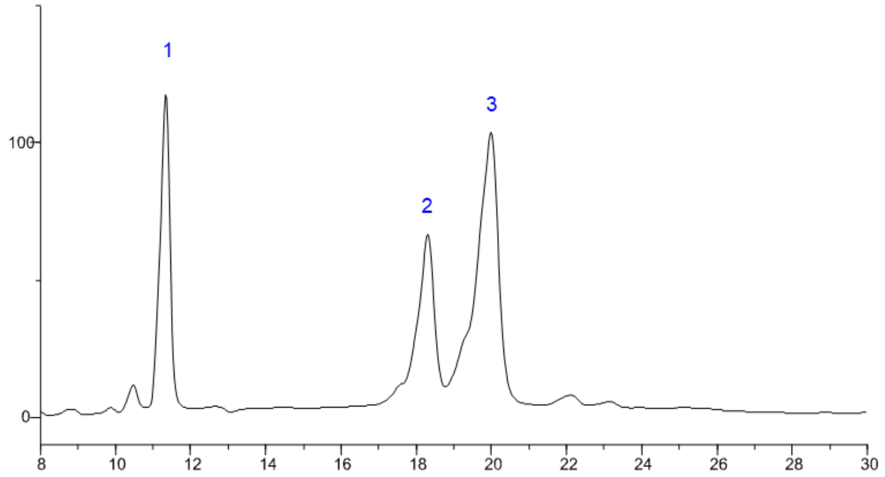

#173 (SA2-TF-Thr)

ESI mass spectrum of HPLC fraction3

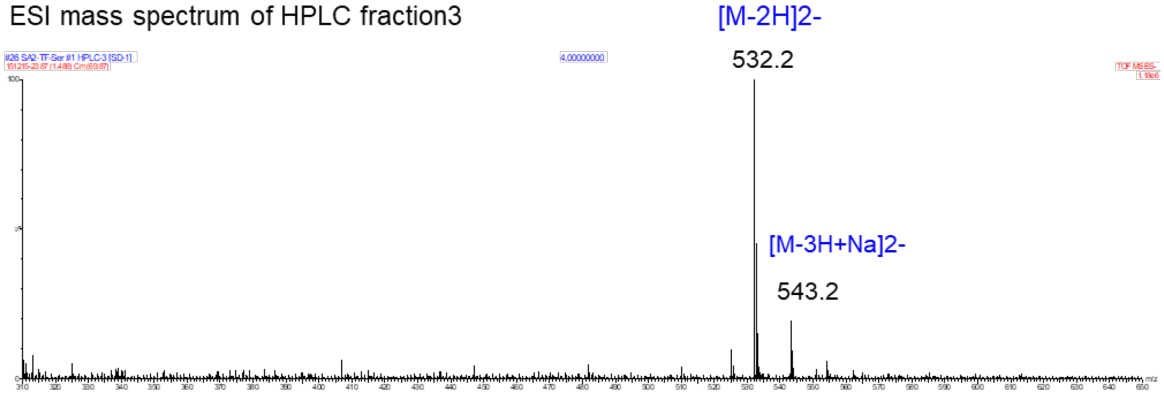

Figure SD1-5. HPLC purification and ESI-MS analysis of sialyl TF antigens.

HPLC profile of a crude STF antigen fraction (F27) from human urine.

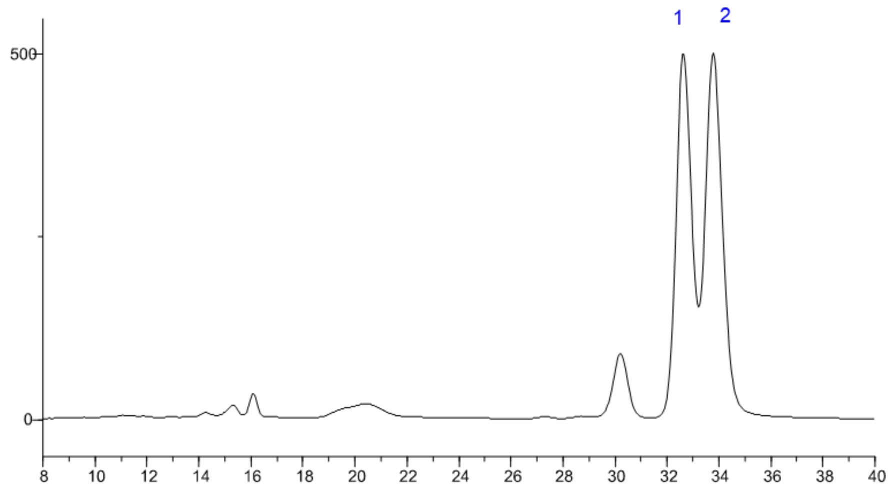

#4 (SA1(2-3)-TF-Ser)

ESI mass spectrum of HPLC fraction 1

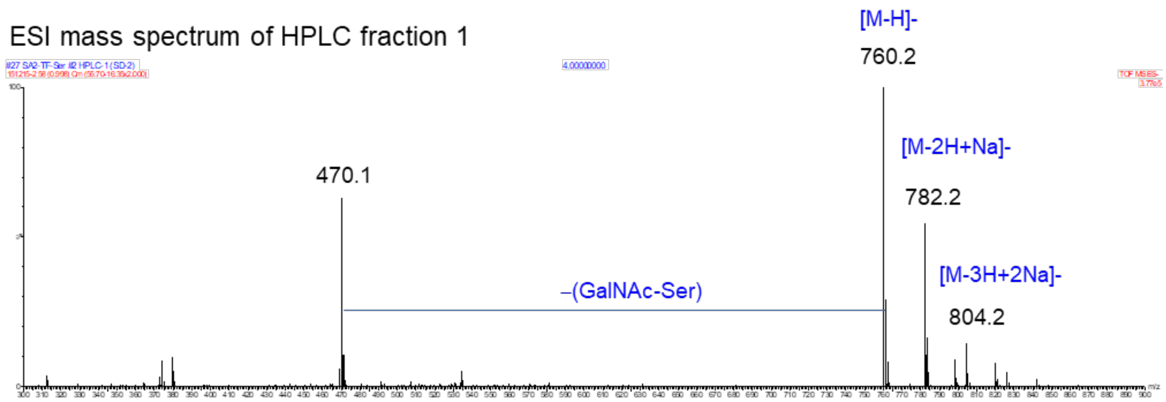

#5 (SA1(2-6)-TF-Ser)

ESI mass spectrum of HPLC fraction 2

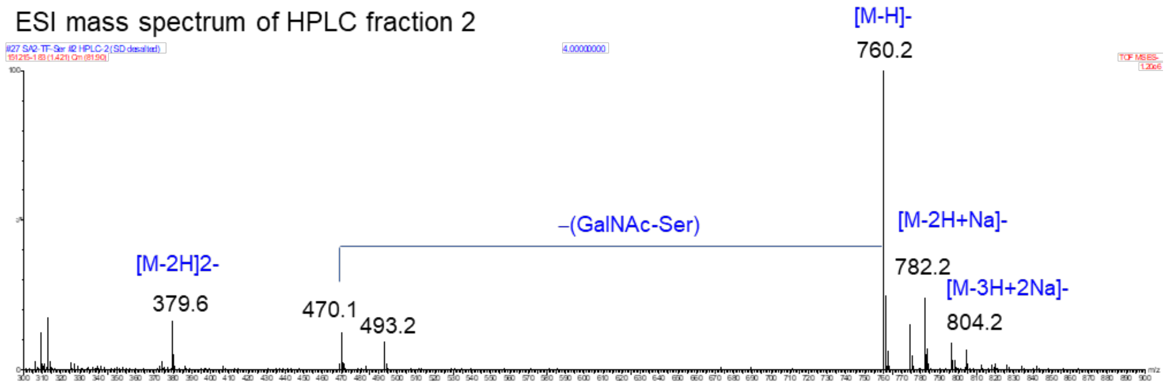

Figure SD1-5 cont. HPLC purification and ESI-MS analysis of sialyl TF antigens.

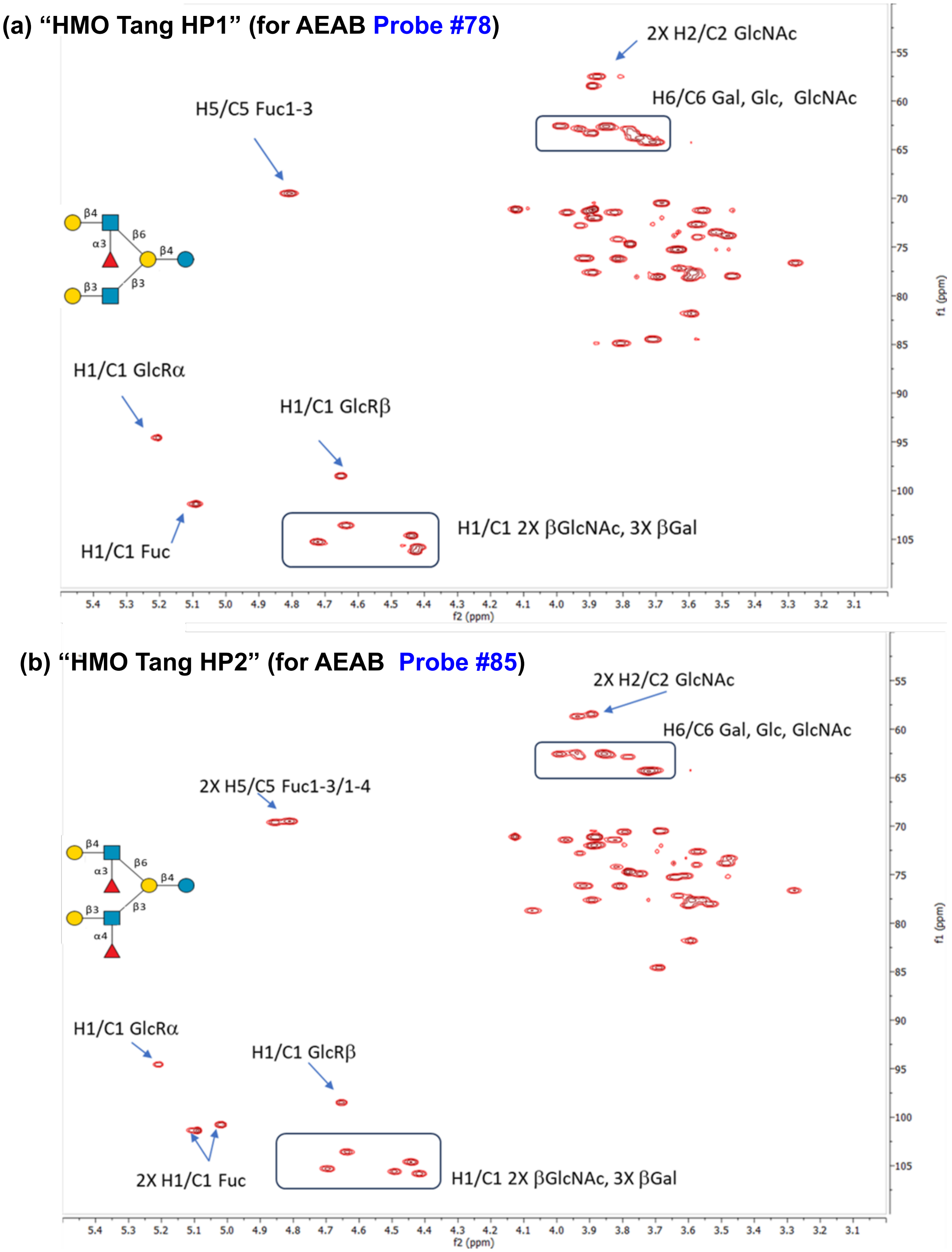

Figure SD1-6. Expanded HSQC NMR spectra for "HMO Tang HP1" (Probe #78) (a) and "HMO Tang HP2" (Probe #85) (b), recorded at 700 MHz, ^{1}H chemical shifts (ppm) are on the horizontal scale and ^{13}C chemical shifts (ppm) are shown on the vertical scale. Some well resolved and informative cross-peaks are labelled: GlcR: reducing end glucose, for which both a- and b-anomers are present.

HPLC separation of N-glycan-AEAB probes derived from bovine fetuin.

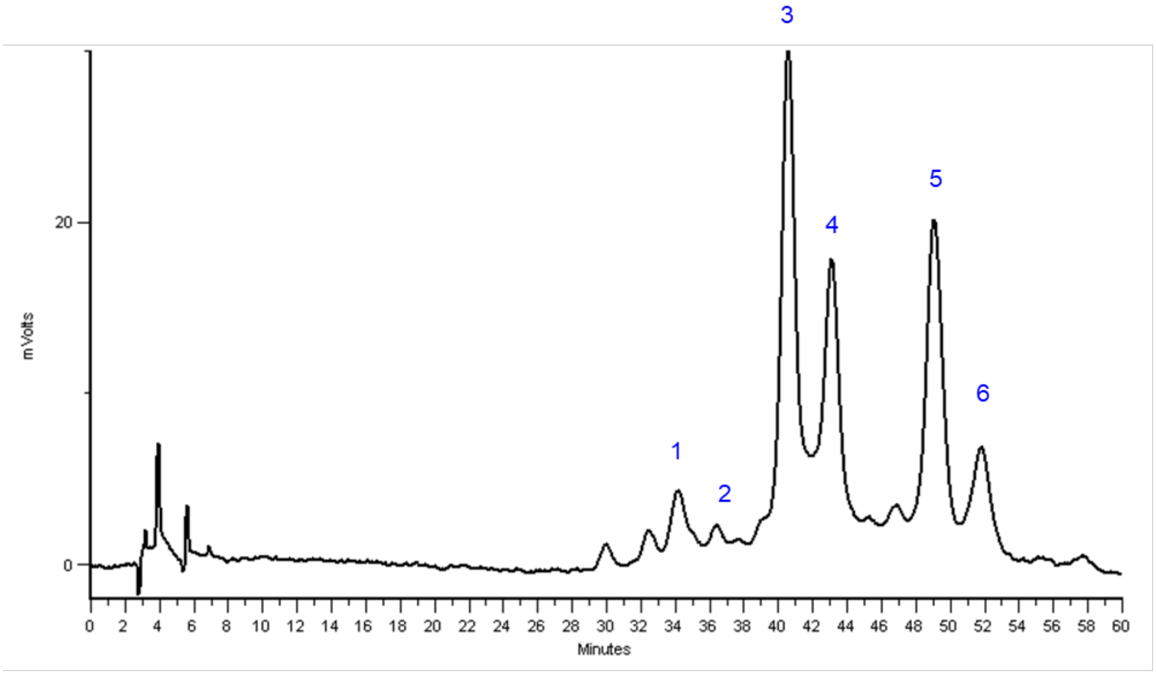

#13 (Fetuin N-glycan A2-AEAB)

ESI mass spectrum of HPLC fraction 1

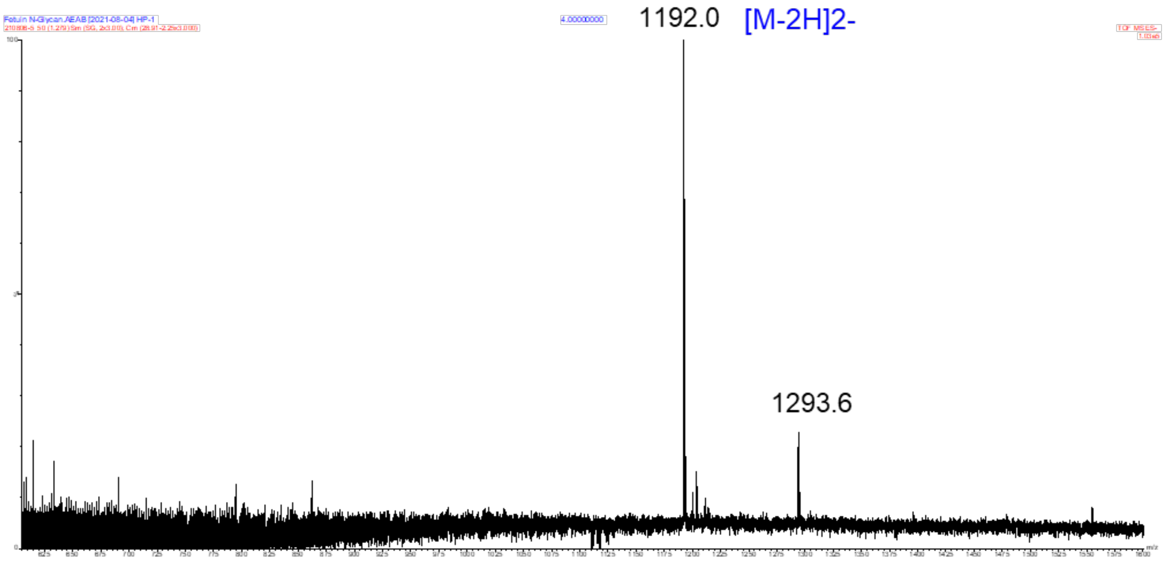

Figure SD1-7. HPLC fractionation and ESI-MS analysis of N-glycans released from bovine fetuin.

#14 (Fetuin N-glycan A3-SA3(a)-AEAB)

ESI mass spectrum of HPLC fraction 5

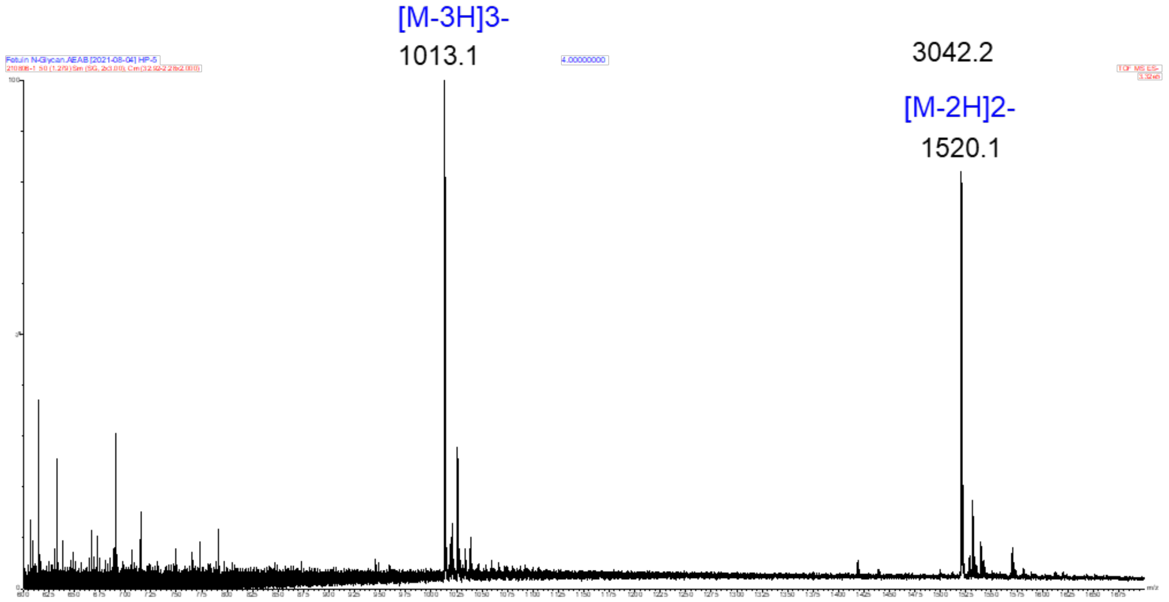

#15 (Fetuin N-glycan A3-SA3(b)-AEAB)

ESI mass spectrum of HPLC fraction 6

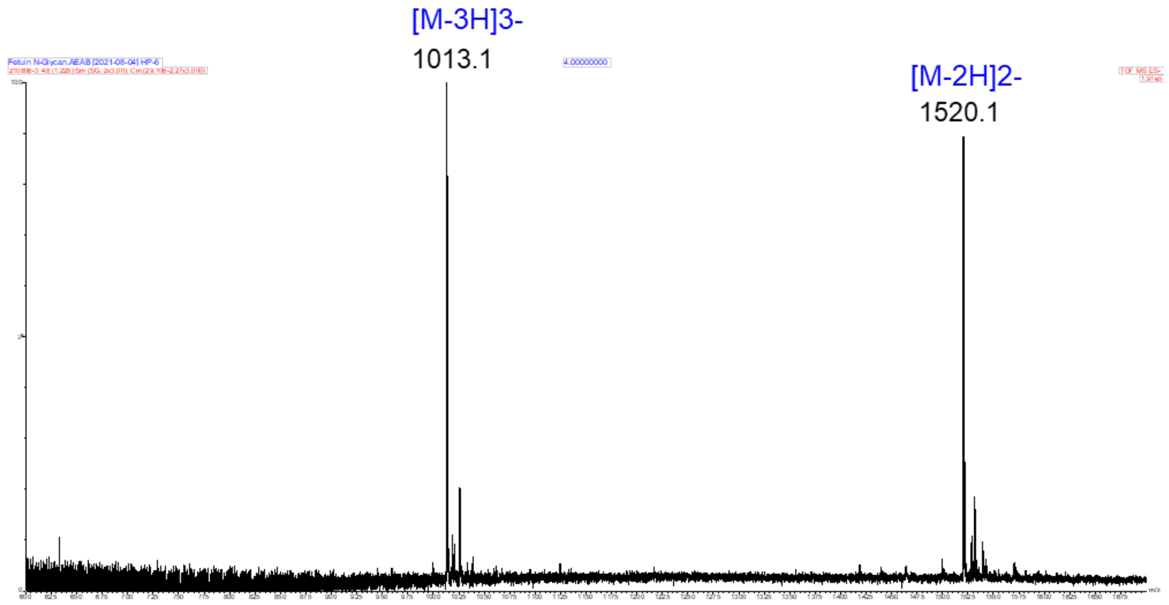

Figure SD1-7 cont. HPLC fractionation and ESI-MS analysis of N-glycans released from bovine fetuin.
